# Novel Antimicrobial Agents for Gram-Negative Pathogens

**DOI:** 10.3390/antibiotics12040761

**Published:** 2023-04-16

**Authors:** Marios Karvouniaris, Maria Panagiota Almyroudi, Mohd Hafiz Abdul-Aziz, Stijn Blot, Elisabeth Paramythiotou, Evdoxia Tsigou, Despoina Koulenti

**Affiliations:** 1 Intensive Care Unit, AHEPA University Hospital, 546 36 Thessaloniki, Greece; karvmarevg@hotmail.com; 2 Emergency Department, Attikon University Hospital, 124 62 Athens, Greece; mariotaalm@yahoo.gr; 3 UQ Centre for Clinical Research, Faculty of Medicine, The University of Queensland, Brisbane, QL 4029, Australia; h.abdulaziz@uq.edu.au (M.H.A.-A.); stijn.blot@ugent.be (S.B.); 4 Department of Internal Medicine and Pediatrics, Ghent University, 9000 Ghent, Belgium; 5 Second Critical Care Department, Attikon University Hospital, 124 62 Athens, Greece; lparamyth61@hotmail.com; 6 Intensive Care Department, ‘Aghioi Anargyroi’ Hospital of Kifissia, 145 64 Athens, Greece; evitsigou@yahoo.com

**Keywords:** Gram-negative, beta-lactamase, carbapenemases, *Acinetobacter baumannii*, *Enterobacterales*, *Pseudomonas aeruginosa*, pharmacokinetics, pharmacodynamics

## Abstract

Gram-negative bacterial resistance to antimicrobials has had an exponential increase at a global level during the last decades and represent an everyday challenge, especially for the hospital practice of our era. Concerted efforts from the researchers and the industry have recently provided several novel promising antimicrobials, resilient to various bacterial resistance mechanisms. There are new antimicrobials that became commercially available during the last five years, namely, cefiderocol, imipenem-cilastatin-relebactam, eravacycline, omadacycline, and plazomicin. Furthermore, other agents are in advanced development, having reached phase 3 clinical trials, namely, aztreonam-avibactam, cefepime-enmetazobactam, cefepime-taniborbactam, cefepime-zidebactam, sulopenem, tebipenem, and benapenem. In this present review, we critically discuss the characteristics of the above-mentioned antimicrobials, their pharmacokinetic/pharmacodynamic properties and the current clinical data.

## 1. Introduction

The spread of multidrug-, extensively drug- and pan drug-resistant Gram-negative bacteria (GNB) in various settings around the world has threatened modern medical practice [1]. The present scarcity of antimicrobial options often makes infections harder to treat. GNB represent four of the six most common pathogens responsible for antimicrobial resistance (AMR) associated mortality (almost a million deaths were attributable to AMR in 2019) [2]. *Enterobacterales* (such as *Klebsiella pneumoniae*, *Escherichia coli*, *Proteus* spp., *Morganella* spp., and *Providencia* spp.) and lactose non-fermenting GNB (such as *Pseudomonas aeruginosa* and *Acinetobacter baumannii*) can produce a wide array of beta-lactamases or enzymes that inactivate many beta-lactam antimicrobials [3]. Beta-lactamases are divided into four classes, A to D: classes A, C, D are serine-based enzymes that inactivate the antimicrobial in two steps, while class B, metallo-beta-lactamases (MBL), employ a metal ion, such as Zn^2+^, for a single step drug hydrolysis [4]. Some of the enzymes can inactivate carbapenems and are called carbapenemases.

Many class A carbapenemases are of the *Klebsiella pneumoniae* carbapenemase (KPC) type; this type contains the globally widespread KPC-2 and KPC-3, with resistome amenable to treatment with modern antimicrobials, such as ceftazidime-avibactam (CAZ/AVI). However, there are less commonly encountered KPC-variants with less favorable susceptibility phenotypes and more challenging treatment choices [5]. Moreover, even KPC-2 and KPC-3 can develop mutations during treatment with diverse mechanisms comprising altered KPCs and non-beta-lactamase resistance mechanisms [6,7]. The members of this class of carbapenemases are numerous and expanding [3].

Metallo-beta-lactamases (MBL) can be either plasmid- or chromosomally encoded. Similar to the serine lactamases, they have been disseminated globally and are the most common class in Asia [8]. Notable class members are the active-on-imipenem (IMP), Verona integron-mediated (VIM), and New Delhi metallo-beta-lactamase (NDM) types of enzymes.

Class D enzymes of the oxacillinase (OXA) type (OXA-23, for example) are associated with *Acinetobacter baumannii* resistance, while OXA-48 are associated with that of *Enterobacterales* resistance [3]. A recent study of *A. baumannii*-related carbapenem-resistance (CR) describes that the addition of novel extrinsic OXA-23 activity to intrinsic oxacillinase OXA-51-like enzyme led to higher minimal inhibitory concentrations (MIC) against imipenem and other beta-lactams, aminoglycosides, and polymyxins [9]. OXA-48-associated resistance is amenable to treatment with CAZ/AVI as avibactam can restore ceftazidime’s antimicrobial activity, while many newly launched beta-lactamase inhibitors, such as vaborbactam and relebactam, cannot reinstall beta-lactam action [10].

Regarding the CR phenotypes, USA data have shown that among 450 carbapenem non-susceptible *Enterobacterales* isolates, a third were non-carbapenemase producers (NCP). The majority of the remaining isolates produced KPC and a few produced MBL enzymes. All the antimicrobials tested showed limited anti-MBL action. However, the respective antimicrobial activity of meropenem/vaborbactam (MER/VARB) and imipenem/cilastatin/relebactam (IMI/CILA/RELE) against NCP exceeded 90% [11]. The significance of the above varies in different geographical regions. Although CR is present globally, the regional differences preclude meaningful clinical comparisons between countries [12]. In addition to the beta-lactamases, many GNB present additional inactivating enzymes to other antimicrobial classes, such as the aminoglycoside-modifying enzymes [13]. Nonetheless, monitoring of resistance through novel microbiological tools, such as whole genome sequencing, may allow better tracking of resistance at a local scale and the discovery of novel resistant traits [14].

Researchers and the industry responded to the challenge by launching novel drugs that are resilient to various resistance mechanisms [15,16,17,18,19,20,21]. Moreover, applying knowledge of the pharmacokinetic/pharmacodynamic (pK/pD) characteristics may optimize antimicrobial drug exposure, leading to maximal therapeutic outcomes. In particular, the treating physicians must modify dosing regimens according to the estimated renal clearance. Dose reduction for all, for example, following the commencement of renal replacement therapy, may result in suboptimal therapeutic levels for many patients [22]. On the contrary, total daily dosing must increase whenever there is augmented renal clearance of the administered antimicrobials [23,24,25]. All the drugs covered in this review, except for eravacycline and omadacycline, are hydrophilic, present a low volume of distribution and protein binding, and are renally cleared [26].

Leading organizations have recently issued recommendations for treating infections associated with difficult-to-treat Gram-negative pathogens. The European Society of Clinical Microbiology and Infectious Diseases (ESCMID) strongly recommends monotherapy for the susceptible microorganism, usually KPC, with either CAZ/AVI or MER/VARB. The Infectious Diseases Society of America (IDSA) recommends monotherapy for KPC infections with either one of the two drugs mentioned above or IMI/CILA/RELE. In the case of MBL-*Enterobacterales*, ESCMID suggests cefiderocol (CEFID) or a combination of two active drugs, as aztreonam-avibactam is currently unavailable. Meanwhile, combining CAZ/AVI with aztreonam is recommended for MBL infections by ESCMID, and IDSA [27,28].

This current review includes novel antimicrobial agents or combinations of existing beta-lactams with novel beta-lactamases inhibitors developed to tackle multidrug-resistant (MDR) GNB, particularly extended beta-lactamase (ESBLs)- and carbapenemase-producing pathogens. Antimicrobial agents active against MDR-GNB approved by the U.S. Food and Drug Administration (FDA) and/or the European Medicines Agency (EMA) since 2017, and agents in advanced development, i.e., in phase 3 clinical trials, are presented and critically discussed. The manuscript focuses on the specific characteristics of the novel antimicrobial agents, their pharmacokinetic/pharmacodynamic properties and the current clinical data. Details on the general characteristics of the antimicrobial classes that the presented antibiotics belong to, is beyond the scope of the current manuscript and for further information we refer the reader elsewhere [29].

### Methods

New drug approvals (and withdrawals) from FDA and EMA were searched in the relevant webpages for the period 2017–2023 [30,31,32,33,34,35,36,37,38,39,40,41,42,43]. Moreover, the World Health Organization (WHO) 2021 Overview and Analysis of Antibacterial Agents in Clinical and Preclinical Development was used to identify agents in advanced development [44], followed by further extensive search in Medline, Clinicaltrials.org, and in the sites of the relevant pharmaceutical companies.

## 2. Recently Approved Antimicrobial Agents

The recently approved antimicrobial agents include cefiderocol that is a new siderophore cephalosporin, imipenem-cilastatin-relebactam and meropenem-vaborbactam that are combinations of a carbapenem with novel beta-lactamase inhibitors, the novel glycylcycline eravacycline, the novel tetracycline omadacycline, and the new tetracycline plazomicin. An overview of the main characteristics of these antimicrobial agents is presented in Table 1.

### 2.1. Cefiderocol

This drug is a modified cephalosporin with a catechol moiety that acts as a siderophore and binds extracellular iron. This drug–iron complex facilitates cefiderocol’s entry into the pathogen cell by both porins and the iron transporter system, whereas it is unaffected by efflux pump overexpression. Thus, high periplasmic space concentrations are achieved. Although its spectrum of activity is broad against Gram-negative pathogens, it lacks activity against Gram-positive and anaerobic microorganisms. Meanwhile, the antimicrobial is stable to hydrolysis by serine class A and class B, and MBL enzymes [45]. Limited data demonstrate stability and clinical efficacy against class D, OXA-48-like carbapenemase-resistant *Enterobacterales* (CRE) isolates [46,47]. In contrast to the above, Poirel et al. studied broad-spectrum beta-lactamase-producing *E. coli* and *P. aeruginosa* isogenic strains. Most MBLs and also some extended-spectrum beta-lactamases (ESBL) showed decreased susceptibility to CEFID [48]. *A. baumannii* can acquire CEFID resistance by producing the ESBL-type beta-lactamase PER-1, which is inactivated in vitro by adding avibactam or durlobactam [49]. Finally, a single resistance marker cannot predict non-susceptibility to CEFID, although combined mechanisms can [50]. Overall, it is primarily helpful for treating difficult-to-treat pathogens, such as extensively drug-resistant (XDR) *Enterobacterales*, *P. aeruginosa*, and *A. baumannii*. CEFID retains activity against most ceftazidime-avibactam- and ceftolozane-tazobactam-resistant *P. aeruginosa* isolates [51].

Regarding microbiological diagnosis, susceptibility interpretation can be challenging, and as such it should be tested with broth microdilution using iron-depleted Mueller–Hinton broth [52]. Moreover, a recent European Committee on Antimicrobial Susceptibility Testing (EUCAST) warning recommends against using commercially available cefiderocol tests and suggests disk diffusion MIC determination instead [52].

#### 2.1.1. Pharmacokinetic-Pharmacodynamic Issues

Cefiderocol demonstrates time-dependent killing characteristics and the pK/pD index associated with optimal activity for *Enterobacterales* and *P. aeruginosa* are on average 73% and 77% of fT > MIC in a thigh murine model [53]. CEFID is dosed according to beta-lactam pK/pD characteristics: (1) it is administered via an extended 3 h infusion; (2) dosing has to be increased to 2 g four times daily (QDS) whenever there is augmented renal clearance (>120 mL/min) [33]; (3) the effluent flow rate may help estimate the dose during continuous renal replacement therapy [54].

The lung penetration ratio was assessed in a recent pharmacokinetic model where the study participants received the regular dosing regimen of 2 g thrice daily (TDS). Considering an MIC of ≤4, the probability of CEFID’s target attainment of time above MIC in the epithelial lining fluid was 87.5% [53].

Gatti et al. measured trough blood levels of CEFID to study the therapeutic target attainment of 13 critically ill patients with ventilator-associated pneumonia (VAP) and bloodstream infections (BSI) by extensively drug-resistant *A. baumannii* treated with CEFID. The fCmin/MIC at steady state was the measure of drug efficacy and considered optimal if it was ≥4, quasi-optimal when between 1 and 4, and suboptimal if <1. The suboptimal ratio was associated with microbiological eradication failure in 80% of the patients [55].

An interesting case report assessed CEFID penetration into the cerebrospinal fluid compartment using two CEFID regimens of 6 and 8 g daily. The drug levels attained were constantly above the minimal inhibitory concentration of the offending pathogen [56].

#### 2.1.2. Clinical Studies

APEKS-NP, a phase 3, multicenter, double-blind, randomized trial, compared the extended, 3 h infusion of 2 g CEFID to that of 2 g meropenem for suspected or proven Gram-negative hospital-acquired pneumonia (HAP), healthcare-associated pneumonia (HCAP), or VAP, while all non-blindly received linezolid for five days [15]. Regarding the primary outcome of overall 14-day mortality, the CEFID group was non-inferior to the control group, as were the secondary outcomes, notably test-of-cure, i.e., clinical cure or microbiological eradication at 7 ± 2 days.

CREDIBLE-CR, in contrast to the previous report, was an open-label randomized study examining CEFID vs. best available therapy (BAT), according to the treating physician’s decision, for severe infections due to CR-GNB [57]. These infections were pneumonia (HAP, HCAP, or VAP), bacteremia, urosepsis, or non-specified sepsis by a suspected or proven CR-GNB. *A. baumannii* was most frequently isolated. The primary study endpoint was clinical cure at day seven following the end of treatment, or eradication of the pathogen in urine, in the case of urosepsis. There were no between-group differences regarding the primary outcome, although the end-of-study (28 ± 3 days) mortality was non-significantly increased in the CEFID group and all-cause mortality was numerically higher in the cefiderocol arm, especially in healthcare-associated pneumonia/HAP/VAP and BSI/sepsis (also at days 14 and 49) [57].

Falcone et al. compared CEFID-containing to colistin-containing regimens in 124 patients with VAP and BSI caused by *A. baumannii*, and performed an inverse probability treatment weighted analysis to examine the effect of the regimen on mortality [58]. In Cox proportional-hazard analysis, the independent predictors for 30-day mortality were septic shock, SOFA score, and age, while receiving CEFID was found protective (hazard ratio 0.32, 95% confidence interval [CI] 0.18–0.57). In subgroup analysis, the effect was confined to BSI, whereas VAP was unaffected by the antibiotic scheme. The colistin group demonstrated higher acute kidney injury rates.

Real-world data on CEFID use for carbapenem-resistant pathogens have recently been published [59]. The authors reported on 172 individuals, including the CREDIBLE-CR patients. Many patients reported adverse events, such as diarrhea or fever, although only four discontinued treatment because of these events [59]. Finally, there is scarce and inconclusive data on the therapeutic potential of combining CEFID with other antibiotics [60].

#### 2.1.3. Approvals

Cefiderocol was approved by FDA in 2019 for complicated urinary tract infection (cUTIs) and in 2020 for HAP/VAP by susceptible pathogens, while in 2020 it was approved by EMA for the same indications.

#### 2.1.4. Molecular Formula

The molecular formula of cefiderocol is C_30_H_34_C_l_N_7_O_10_S_2_. For the 2D structure of cefiderocol, please see Figure S1 of the Supplementary material.

### 2.2. Imipenem-Cilastatin-Relebactam

Relebactam is a novel beta-lactamase inhibitor that potently inhibits class A and class C beta-lactamases. Although chemically related to avibactam, it does not add to imipenem activity against OXA-48 [61]. The drug is formulated as a triple combination—apart from imipenem and relebactam, it contains the renal dehydropeptidase-I inhibitor cilastatin. Although the inhibitor renders most imipenem-non-susceptible KPC-producing *Enterobacterales* and *P. aeruginosa* isolates susceptible, the metallo-proteinase-producing strains remained non-susceptible to the combination [62]. The combination of IMI/CILA/RELE plus aztreonam was tested against MBL-producing OprD-deficient *P. aeruginosa* isolates and it presented synergy [63]. IMI/CILA/RELE can retain activity against many ceftazidime-avibactam- and ceftolozane-tazobactam-resistant *P. aeruginosa* isolates [51,64].

Moreover, another research group performed time-kill analysis of 13 *K. pneumoniae* and *E. coli* isolates, co-harboring MBL and serine beta-lactamases and found that they were resistant to IMI/CILA/RELE (100%) and aztreonam (85%), when each antibiotic was given alone. However, when combined, they were synergistic against 11/13 of the isolates [65]. E-test may be used for observing synergy, although the data are premature [66].

Finally, the intrinsic resistance of the family *Morganellaceae* (*Proteus* spp., *Morganella* spp., and *Providencia* spp.) to imipenem is unaffected by the inhibitor [67,68].

#### 2.2.1. Pharmacokinetic-Pharmacodynamic Issues

Unbound relebactam 24 h area under the concentration time curve to IMI/CILA/RELE MIC (fAUC/MIC) can best prognosticate the drug’s antimicrobial efficacy. A ratio value of 7–7.5 can predict a 2-log10 kill of *P. aeruginosa* strains, based on hollow fiber, and a neutropenic murine thigh model [69].

Population pK/pD modeling, based on clinical study data, confirmed that the renal function-based recommended scheme is sufficient for treatment [70]. The recommended dose for normal renal function dose, 1.25 g four times daily infused over 30 min, could achieve a probability of target attainment (PTA) of 93% at a MIC ≤ 2, even in the case of augmented renal clearance (>130 mL/min) [24].

An in vitro pD model used simulated unbound human plasma levels of IMI/CILA/RELE corresponding to the recommended dose of 1.25 QDS, alone or in the presence of colistin or amikacin. IMI/CILA/RELE plus colistin resulted in colony-forming unit (CFU) reductions in some of the studied *P. aeruginosa* isolates, indicative of either synergy or additivity, whereas adding amikacin did not increment the antibacterial IMI/CILA/RELE action [71].

#### 2.2.2. Clinical Studies

RESTORE-IMI 1, a phase 3 multicenter, double-blind trial, studied patients with infections, most commonly urinary tract infection or pneumonia, due to imipenem non-susceptible pathogens. The primary endpoint was a favorable response according to predefined criteria for every infection type [19]. The microbiologically modified intention-to-treat population comprised 31 individuals. *P. aeruginosa* (77.4%) predominated in this cohort. The intervention group received IMI/CILA/RELE, while the control group received imipenem plus colistin. The favorable 28-day clinical response was 71.4% vs. 40%, whereas the respective mortality was 9.5% vs. 30% (both outcomes were non-significantly different). Concerning adverse events in the safety population (*n* = 47), the treatment-emergent nephrotoxicity was 10.3% vs. 56.3% (*p* = 0.002).

RESTORE-IMI 2 was a phase 3, HAP/VAP, multicenter, double-blind trial comparing IMI/CILA/RELE to piperacillin/tazobactam. Two-thirds of the patients were in the ICU, half ventilated, while the most isolated bacteria were *K. pneumoniae* and *P. aeruginosa*. The modified intention-to-treat population included 531 patients that had received at least a single drug dose and did not solely present Gram-positive cocci at the baseline stain [20]. The study group was non-inferior compared to the control group regarding the primary trial endpoint, i.e., overall mortality, and the key secondary endpoint of favorable clinical response at early follow-up (on days 7–14 following the end of treatment). Meanwhile, the adverse events were similar in both groups.

Kohno et al. evaluated the efficacy and adverse effect profile of IMI/CILA/RELE, in a multicenter, observational non-comparative phase 3 study of 81 patients with cUTIs and complicated intra-abdominal infections (cIAIs). *E. coli*, with favorable resistance phenotype, predominated in this cohort [72]. The drug-related adverse effects were overall 18.5% and primarily infusion-related. The efficacy was excellent and over 80% at the end of treatment.

Rebold et al. examined IMI/CILA/RELE use in 21 more severely ill patients than in the RESTORE-IMI trials. Out of the 21 patients, 16 were in the ICU, with a median APACHE II score of 21.5. Pneumonia, either HAP or VAP, occurred in 52% of individuals, and the predominant microorganism isolated was MDR *P. aeruginosa*. The all-cause 30-day mortality was 33% [73].

#### 2.2.3. Approvals

Imipenem-cilastatin-relebactam was approved by FDA in 2019 for cUTIs and cIAIs, and 2020 for HAP/VAP by susceptible pathogens, while in 2020 it was approved by EMA for HAP/VAP and associated (or suspected to be associated) bacteremia and GNB infections with limited treatment options.

#### 2.2.4. Molecular Formula

The molecular formula of imipenem is C_12_H_17_N_3_O_4_S, of cilastatin is C_16_H_26_N_2_O_5_S and of relebactam is C_12_H_20_N_4_O_6_S. For the 2D structures, please see Figure S2 of the Supplementary material.

### 2.3. Meropenem-Vaborbactam

Vaborbactam, a non-antibiotic cyclic boronic acid inhibitor, binds reversibly with meropenem. The complex improves meropenem’s stability to class A and C beta-lactamases; however, it is inactive against class B and D lactamases [61]. The inhibitor cannot improve the susceptibility of *P. aeruginosa* to meropenem due to the commonly present NCR, and *A. baumannii* due to the presence of class B and D beta-lactamases [74].

Regarding HAP/VAP isolates, the drug was active against >90% of KPC-producing *Enterobacterales* and 70–80% of *P. aeruginosa* [74,75]. KPC-producing *K. pneumoniae* strains that develop resistance to CAZ/AVI often leads to MER/VARB cross-resistance through increased KPC expression, although some isolates remained susceptible to MER/VARB [76,77]. Indeed, MER/VARB retained the ability to better interact with KPC-2 variants than ceftazidime-avibactam; however, resistance selection was primarily due to NCR [77,78,79].

#### 2.3.1. Pharmacokinetic-Pharmacodynamic Issues

The vaborbactam fAUC to MER/VARB MIC correlated with antimicrobial activity, while a ratio value of 36 in the hollow fiber infection model can be bactericidal [61]. MER/VARB is primarily renally cleared, and dosing adjustments are needed for patients with renal impairment. Bhavnani et al. performed pK/pD target attainment analyses of the drug’s 2 g/2 g dose and the modified dosing that corresponds to impaired renal function. The recommended dosing regimen achieved the target for treating *P. aeruginosa* and *Enterobacterales* over 80% of the time at an MIC value of 4–8 mg/L [80]. Kufel et al. measured in vivo, in a single patient, the pre-and post-filter levels during continuous renal replacement therapy and found that a dosing schedule of 1 g/1 g TDS may suffice to achieve drug concentrations above the MIC of the pathogen [81].

#### 2.3.2. Clinical Studies

TANGO II, a phase 3, open-label, multicenter study, was prematurely stopped at the prespecified interim analysis as its objectives were met and finally included 77 individuals. MER/VARB was compared to the best available therapy (ceftazidime/avibactam monotherapy or mono-/combination treatment of the following antimicrobial options: colistin, meropenem, aminoglycosides, and tigecycline). The microbiologic-carbapenem-resistant-*Enterobacterales* modified intention-to-treat population, which received at least a single drug dose, included 47 patients. Bacteremia was the most common diagnosis, while KPC-producing *K. pneumoniae* predominated. The study endpoints in the above population were as follows: (1) cure at the end of treatment; (2) test-of-cure at day 7 ± 2 later; and (3) 28-day overall mortality. The respective results were in favor of MER/VARB 65.6% vs. 33.3%, 59.4% vs. 26.7% (both significant), and 15.6% vs. 33.3% (non-significant) [18].

Real-world data on CRE infections (40% were BSI) were reported by Ackley et al. in a multicenter retrospective cohort study of 131 (57.3% critically ill) participants. The dominant mechanism of CR (~75%) was KPC production. The authors compared MER/VARB to CAZ/AVI. Less CAZ/AVI-treated patients received combination therapy with other antimicrobials than MER/VARB patients. Clinical success, a composite outcome of clinical and microbiological favorable endpoints, did not differ between the groups, which also had similar adverse event rates [82].

Alosaimy et al. provided more real-world data about MER/VARB use for CRE and CR *P. aeruginosa* infections. One-hundred twenty-six adult patients were retrospectively studied. They presented significant comorbidities, and half had been ICU residents. They received MER/VARB for at least 72 h and suffered suspected or confirmed MDR-GNB infection. The adverse clinical outcomes of death or readmission on day 30 was significantly lower in the patients who appropriately received MER/VARB earlier (≤48 h from infection onset) than those who received it later (17.6% vs. 38.6%). The adverse rate was low, at 3.2% [83].

Finally, in a retrospective multicenter Italian study, 37 patients with MER/VARB sensitive, KPC-*K. pneumoniae*, infections were treated compassionately with MER/VARB for at least 72 h. Notably, 51.9% of the isolates were CAZ/AVI-resistant. The bloodstream and the lower respiratory tract were the most frequent infection locations. Most patients were critically ill while treatment followed infection onset by five days. The overall hospital mortality was 24.3%. It did not differ between the ceftazidime-resistant and ceftazidime-sensitive subgroups. Recurrent BSI was evident in just three patients after 18 days (median) of treatment, although the isolates remained susceptible to the drug. These patients achieved clinical cure after they received a combination of MER/VARB with a second antimicrobial (colistin or fosfomycin) [84].

#### 2.3.3. Approvals

Meropenem-vaborbactam was approved by FDA in 2017 for cUTIs, including pyelonephritis, and by EMA in 2018 for cUTIs, cIAIs, HAP/VAP, bacteremia associated or suspected to be associated with any of the listed infections, and infections by GNBs with limited treatment options.

#### 2.3.4. Molecular Formula

The molecular formula of meropenem is C_17_H_25_N_3_O_5_S and of vaborbactam is C_12_H_16_BNO_5_S. For the 2D structures, please see Figure S3 of the Supplementary material.

### 2.4. Eravacycline

Eravacycline (ERA) is a novel glycylcycline that shares a similar antibacterial spectrum of activity with the other family member, tigecycline. This spectrum covers Gram-positive, Gram-negative, and anaerobic microorganisms, including MDR strains. However, the MIC is 2- to 4-fold less than that of tigecycline. Notably, ERA is inactive against *P. aeruginosa* [85].

#### 2.4.1. Pharmacokinetic-Pharmacodynamic Issues

Eravacycline’ efficacy against *Enterobacterales* was examined with a murine thigh infection model. An fAUC/MIC ratio mean (±SD) of 5.6 (±5) was associated with a 1-log isolate reduction, while the peak free drug concentration to MIC ratio was correlated with the efficacy [86].

In a phase 1 pK study of ERA, the penetration of the drug into the epithelial lining fluid and alveolar macrophages were evaluated. Interestingly, the levels were 6-fold and 50-fold, respectively, compared to plasma. Therefore, ERA may become an alternative option for treating pneumonia caused by difficult-to-treat GNB [87].

#### 2.4.2. Clinical Studies

IGNITE 1 and IGNITE 4 were the trials that assessed the drug in patients with cIAI who also had an operation or percutaneous drainage within 48 h of diagnosis [16,17].

IGNITE1 was a double-blind, multicenter, non-inferiority trial that compared eravacycline 1mg/kg twice daily (BD) to ertapenem 1 g once daily (OD) for treating hospitalized patients with cIAIs, at least for four days. The primary outcome and clinical cure rate, 25–31 days following the start of treatment, for the microbiological intention-to-treat population, was 86.8% vs. 87.6% [16].

IGNITE4 was a randomized, double-blind, multicenter, non-inferiority (margin of 12.5%) trial of complicated intraabdominal infections in the hospital. It compared ERA 1mg/Kg BD to meropenem 1 g TDS. The overall primary outcome was 90.8% vs. 91.2% for the ERA and meropenem groups, respectively. ERA was not inferior to MER concerning the primary endpoint of infections by ESBL-producing *Enterobacterales*, which were 87.5% vs. 84.6%. The safety profile of ERA was favorable, with class-effect low rates of nausea, vomiting, and diarrhea [17]. Despite the few *A. baumannii* isolates of the above studies, there were all susceptible to ERA.

A Bayesian network meta-analysis compared ERA to seven other commonly administered antimicrobials, including tigecycline, for treating cIAIs. The efficacy and safety of ERA were like the competing drugs. However, in microbiological response, ERA fared better than tigecycline [88].

A meta-analysis of three studies, including IGNITE1 and IGNITE4, showed that ERA was an effective clinical alternative to carbapenem treatment. The nausea rate was increased, although severe adverse effects did not differ between ERA and the carbapenem comparator [89].

Finally, Alosaimy et al. have provided real-world data about treating *A. baumannii* infections (69.5% of the isolates were CR) with ERA. Most patients received combined antimicrobials, while the most frequent diagnosis was pneumonia. Thirty-day mortality was 23.9 (21.9)%, implying that ERA could be another option for treating these challenging infections, which may present mortality in excess of 40%, in the case of BSI and HAP/VAP [90,91].

#### 2.4.3. Approvals

Eravacycline was approved in 2018 by both FDA and EMA for cIAIs.

#### 2.4.4. Molecular Formula

The molecular formula of eravacycline is C_27_H_31_FN_4_O_8_. For the 2D structure of eravacycline, please see Figure S4 of the Supplementary material.

### 2.5. Omadacycline

Omadacycline (OMA) is a novel aminomethylcycline, a new drug that belongs to the tetracycline class. It demonstrates activity against a broad-spectrum of Gram-positive, Gram-negative, atypical and anaerobic bacteria. Regarding anti-Gram-negative activity, its spectrum includes many members of the *Enterobacterales* family, except for *Proteus*, *Providencia*, and *Morganella* species. Almost half of the carbapenem-non-susceptible *Enterobacterales* isolates remain susceptible to OMA [92]. Although OMA lacks activity against *Pseudomonas* spp. [40,41], it retains activity against some *A. baumannii* isolates [93]. The therapeutic potential of OMA against *A. baumannii* isolates, regardless of their susceptibility to minocycline, was examined in a recent study [94]. The combination of OMA with sulbactam was promising as it showed synergy against 80% of the isolates [94].

#### 2.5.1. Pharmacokinetic-Pharmacodynamic Issues

The in vitro OMA pD indices for five *A. baumannii* and five *E. coli* strains have been recently assessed. Notably, the relevant index for tetracycline action is the fAUC/MIC. The respective ratios for a 24 h static effect were 108.1 ± 38.6 and 25.3 ± 17.2. Meanwhile, a single dose of 100 mg IV, the licensed daily scheme, resulted in an AUC of 10 mg/L * h [41,95]. Thus, the recommended dose fell short of achieving meaningful anti-Gram-negative activity. Regarding CR *A. baumannii*, higher in vitro exposures simulating 200 mg and 400 mg were tested and were not bactericidal alone; however, these higher exposures presented synergy with meropenem against 3 out of 8 isolates [96].

About human studies, a pK study compared the steady-state concentrations of omadacycline to those of tigecycline in the epithelial lining fluid (ELF) and the alveolar cells (EC). The OMA ratios of ELF/total plasma and EC/total plasma levels were higher for OMA than tigecycline at the dosing schedule recommended by the summary of product characteristics. Meanwhile, OMA unbound plasma levels surpassed those of tigecycline and ERA [97].

#### 2.5.2. Clinical Studies

There is no clinical study of OMA for the treatment of MDR-GNB.

#### 2.5.3. Approvals

Omadacycline was approved in 2018 by FDA for acute bacterial skin and skin structures infections (ABSSSIs) and community-acquired bacterial pneumonia (CABP). The drug application was withdrawn from the EMA [40,41].

#### 2.5.4. Molecular Formula

The molecular formula of omadacycline is C_29_H_40_N_4_O_7_. For the 2D structure of omadacycline, please see Figure S5 of the Supplementary material.

### 2.6. Plazomicin

This novel semi-synthetic aminoglycoside was highly active in vitro against most *Enterobacterales*, *P. aeruginosa*, and *A. baumannii* isolates at an MIC ≤ 4. The antimicrobial could inhibit most gentamycin, tobramycin, and amikacin-resistant isolates [98]. Moreover, the drug retains in vitro activity against two-thirds of CR isolates, more than MER/VARB and OMA [99]. Of interest, plazomicin may remain active against MBL-producing Enterobacterales. However, CRE can produce 16S rRNA methyltransferase, harbored into plasmids which invariably abrogates aminoglycoside use [100,101].

Regarding the susceptibility testing of Enterobacterales to plazomicin, a novel gradient diffusion method (ETEST Plazomicin) was compared to the standard broth microdilution method. The essential between-the-methods agreement was 99% [102].

#### 2.6.1. Clinical Studies

In a multicenter trial of 609 patients, 15 mg/Kg PLAZ, once daily, was compared to MER 1g TDS to treat cUTIs and acute pyelonephritis. The primary endpoint was the composite cure (patients were clinically cured and bacteria were eradicated) on day 5 and days 15 and 19 following therapy commencement (test-of-cure visit) in the microbiologic modified intent-to-treat population (those patients who had a positive urine culture and received at least a study drug dose). PLAZ was non-inferior to MER, while more patients at the test-of-cure visit presented microbiological eradication [103].

Combating Antibiotic-Resistant Enterobacteriaceae (CARE) trial compared PLAZ to COLI for treating BSI and HAP/VAP due to suspected or confirmed CRE. It was prematurely terminated due to poor enrollment. The microbiologically modified intention-to-treat population comprised 37 patients (28 BSI and 9 pneumonia). PLAZ-treated individuals presented lower mortality from day 14 through day 60. The adverse effects were less frequent in the PLAZ (50%) than in the COLI group (81%) [21].

A meta-analysis of three studies (including the above two) confirmed the superiority of PLAZ to the competitors concerning microbiologic recurrence. However, the drug’s clinical efficacy for CRE-associated BSI and pneumonia remains undetermined [104].

#### 2.6.2. Approvals

Plazomicin was approved by FDA in 2018 for cUTIs. It is not available in Europe, as the marketing company withdrew its application in 2020 [43].

#### 2.6.3. Molecular Formula

The molecular formula of plazomicin is C_25_H_48_N_6_O_10_. For the 2D structure of plazomicin, please see Figure S6 of the Supplementary material.

## 3. Antimicrobials in Phase 3 Clinical Trials

Antimicrobial agents active against MDR-GNB, currently in phase 3 clinical trials include combinations of existing beta-lactams with novel beta-lactamase inhibitors, namely, aztreonam-avibactam, cefepime-enmetazobactam, cefepime-taniborbactam, cefipime-zidebactam; and novel antibiotics, namely, the novel carbapenems sulopenem, tebipenem, and benapenem. An overview of the main characteristics of these antimicrobial agents is presented in Table 2.

### 3.1. Aztreonam/Avibactam

The drug combines aztreonam’s inherent stability to MBLs and avibactam’s inhibition of class A and C beta-lactamases. Many CRE strains co-produce MBLs and serine enzymes that can inactivate aztreonam. Indeed, a beta-lactamase inhibitor, such as avibactam, zidebactam, or nacubactam, protects aztreonam from hydrolysis and enhances its anti-MBL activity towards Enterobacterales and, to a lesser extent, towards *P. aeruginosa* [105,106]. The addition of the already commercially available combination of CAZ/AVI could restore aztreonam’s activity against MBL-producing *Enterobacterales*, while *Stenotrophomonas maltophilia* was 100% susceptible to the triple combination, though the tested isolates were limited [107]. A recent large in vitro broth microdilution study confirmed the potent activity of AZT/AVI against *Enterobacterales* isolates. Notably, all studied CPE producers were inhibited using a concentration threshold of 8 mg/L [108]. Moreover, combined ceftazidime/avibactam/aztreonam was synergetic at inhibiting four out of five *P. aeruginosa* isolates [109]. Finally, AZT/AVI possesses a broader spectrum than CAZ/AVI; however, an *E. coli*-KPC-21-producing strain was resistant to the former while remaining susceptible to the latter combination [110,111].

#### 3.1.1. Pharmacokinetic-Pharmacodynamic Issues

Smith et al. examined, in vitro and in vivo, an NDM-1 and a CTX-M co-producing *K. pneumoniae* infection using hollow fiber and neutropenia rabbit models. They studied the sequelae by adding polymyxin B to the aztreonam-CAZ/AVI combination. In the presence of polymyxin B, they demonstrated enhanced bacterial killing and reduced inflammation, while suppressing resistance [112].

Aztreonam, co-administered with CAZ/AVI, was studied in healthy subjects which resulted in reduced AZT clearance. Meanwhile, continuously infused AZT was associated with severe hepatic transaminase elevations [113].

A phase 2 open-label multicenter study of AZT/AVI for cIAIs compared three dosing regimens for pK/pD target attainment. The regimen that prevailed and presented no safety concerns was the 500 mg/167 mg loading dose followed by the 1500 mg/500 mg QDS, as a 3 h infusion [114].

#### 3.1.2. Clinical Studies

A phase 3 study of AZT/AVI vs. best available treatment for severe infections due to MBL-producing bacteria is still recruiting participants (NCT03580044) [115].

In the meantime, combining a beta-lactam/beta-lactamase inhibitor with aztreonam is viable for MBL-targeted treatment. A recent systematic review summarized the CAZ/AVI-avibactam’s clinical use against MBL-producing pathogens [105]. Ninety-four patients, 83% with bacteremia, were administered the combination, almost exclusively (99%) as targeted therapy. Two studies that included data from 128 patients, half with this combination vs. half with other active treatments, were meta-analyzed. The pooled crude 30-day mortality was 19% vs. 44%, (odds ratio 0.33, 95% [CI] 0.13–0.66); however, the mortality of the comparator group is driven by colistin-containing regimens, where 21 out of 36 patients died [105].

#### 3.1.3. Molecular Formula

The molecular formula of aztreonam is C_13_H_17_N_5_O_8_S_2_ and of avibactam is C_7_H_11_N_3_O_6_S. For the 2D structures, please see Figure S7 of the Supplementary material.

### 3.2. Cefepime/Enmetazobactam

Cefepime/enmetazobactam (CEFI/ENM) is a β-lactam/β-lactamase inhibitor combining cefepime, a fourth-generation cephalosporin, with enmetazobactam (formerly AAI101), a novel extended-spectrum β-lactamase inhibitor. Enmetazobactam structurally resembles tazobactam but possesses a methyl group on the triazole ring that enhances its activity against β-lactamases. Cefepime’s susceptibility rates are expanded from 2% to 98% with the combination of enmetazobactam, which exhibits potent activity against class A ESBL-producing bacteria [116]. Enmetazobactam only weakly inhibited class D OXA-10 and OXA-48 and moderately inhibited the class C β-lactamase. MBLs, including NDM-1, are not inhibited by enmetazobactam [116]. Although some activity against KPCs is reported in the literature, these results have not been confirmed by other studies, and currently, CEFI/ENM cannot be used against carbapenem non-susceptible strains [116,117,118,119]. It has no activity against *A. baumannii* and *S. maltophilia* and should not be used against AmpC-producing *Pseudomonas* strains [116]. Notably, enmetazobactam addition did not alter the efficacy of cefepime against *P. aeruginosa* isolates collected in the US and Europe, as the MIC_90_ for both cefepime and CEFI/ENM was 16 μg/mL [118].

#### 3.2.1. Pharmacokinetic-Pharmacodynamic Issues

The pharmacokinetic-pharmacodynamic index that was best associated with potency against ESBL-producing isolates of *K. pneumoniae* was the time above free threshold concentration, fT > CT [120]. Specifically, 44% fT > 2 μg/mL of enmetazobactam was needed for 1-log10 bioburden reduction, combined with a cefepime pK/pD target of 40 to 60% fT > CEFI/ENM MIC [120]. The dosing regimen supported in the literature is 2 g/500 mg of CEFI/ENM administered every 8 h [119,120,121]. A phase 1 clinical trial has completed recruitment, and results are expected concerning the pK parameters and safety of the combination in patients with various degrees of renal impairment (NCT03680352) [122].

Cefepime and enmetazobactam exhibited adequate penetration into the epithelial lining fluid, with AUC plasma: AUC ELF ratios of 61% and 53%, respectively, in healthy subjects, while ELF exposure estimates of both combination components were strongly correlated [121]. In a neutropenic murine pneumonia model, the AUC ELF: AUC plasma ratios were 73% and 62%, respectively [123].

#### 3.2.2. Clinical Studies

The combination has been studied in a phase 3 clinical trial (ALLIUM trial), where CEFI/ENM was non-inferior to piperacillin/tazobactam in treating cUTIs. The study was superior to the control drug in terms of clinical cure and microbiological eradication; the overall success rate was 79.1% vs. 58.9%, (95% [CI] 14.3%–27.9%) [124]. CEFI/ENM was administered 2 g/500 mg TDS as a 2 h infusion. Interestingly, *Clostridioides difficile* infections were noted more often in CEF/ENM group [124].

By the end of 2022, a marketing authorization will be submitted to EMA, while the FDA has granted CEF/ENM with qualified Infectious Disease Product and Fast Track designation. Regarding HAP and VAP, EMA has allowed the company to seek approval without conducting a Phase 3 study with this indication, based on adequate intrapulmonary penetration of the drug into the ELF [121,124].

In summary, cefepime/enmetazobactam is a better option for treating cUTIs than piperacillin/tazobactam and has a potential role as a carbapenem-sparing regimen because it possesses excellent activity against ESBL-producing *Enterobacterales*.

#### 3.2.3. Molecular Formula

The molecular formula of cefepime is C_19_H_24_N_6_O_5_S_2_ and of enmetazobactam is C11H14N4O5S. For the 2D structures, please see Figure S8 of the Supplementary material.

### 3.3. Cefepime/Zidebactam

In addition to being a novel β-lactamase inhibitor, zidebactam (WCK 5107) is also active against beta-lactamase when it binds to Penicillin Binding Protein 2 (PBP2) of *Enterobacterales* and *P. aeruginosa* [125]. The simultaneous inactivation of PBP2 and PBP3 by zidebactam and cefepime confers a strong and rapid bactericidal potential to the combination. Zidebactam is active in vitro against a wide range of β-lactamases from classes A, B, C, and D, including KPC, MBLs, and OXA-48 carbapenemases [126]. The MIC_50_ and MIC_90_ values for cefepime/zidebactam (CEFI/ZIDE) against carbapenemase-producing isolates were 0.5 and 16 μg/mL, respectively, showing enhanced activity compared with other commonly used agents (imipenem, amikacin, polymyxin B, ceftolazone-tazobactam, and piperacillin-tazobactam). The MIC_50_ and MIC_90_ values against class B-producing- and class D-producing-carbapenemases were 2 and 32 μg/mL, and 2 and 16 μg/mL, respectively [127]. In another study, 86.1% of the CRE strains and 89.8% of CR-*K. pneumoniae* isolates were inhibited by CEFI/ZIDE at 2 mg/L [128]. In contrast, most CRE and CR-*P. aeruginosa* isolates were susceptible to CEFI/ZIDE [129]. At MICs ≤ 8 mg/L, only 34% of CR-*A. baumanii* isolates were sensitive to CEFI/ZIDE [130]. Additionally, CEFI/ZIDE has shown promising activity against *S. maltophilia* and *Burkholderia cepacia* [131].

#### 3.3.1. Pharmacokinetic-Pharmacodynamic Issues

CEFI/ZIDE demonstrates time-dependent killing characteristics and the fT > MIC is the pK/pD index associated with efficacy [132]. CEFI/ZIDE is administered 2 g/1 g every 8 h, as an 1 h infusion, and dose adjustment is required for patients with renal impairment as the drug is renally excreted [133].

In a neutropenic mouse *A. baumannii* lung infection model, the addition of zidebactam to cefepime achieved the 1-log_10_ kill endpoint with a reduced fT > MIC as low as 15.5% [134]. Similarly, adding zidebactam to cefepime in a neutropenic mouse *Enterobacterales* pneumonia model achieved a target of 31% T > MIC and the 1-log10 endpoint [132].

Regarding pneumonia treatment potential, the total plasma/ELF penetration ratio for CEFI/ZIDE ranged from 0.3 to 0.5 [134], while in another study, the penetration into ELF was 50% and 70% for cefepime and zidebactam, respectively [132].

In vivo efficacy of CEFI/ZIDE has been shown in neutropenic murine lung and thigh infection models against CR-*A. baumannii* and MDR-*P. aeruginosa* [135,136].

#### 3.3.2. Clinical Studies

Currently, a phase 3 clinical trial (NCT04979806) is recruiting patients to compare cefepime-zidebactam to meropenem for treating hospitalized adults with cUTIs or acute pyelonephritis [137].

#### 3.3.3. Molecular Formula

The molecular formula of cefepime is C_19_H_24_N_6_O_5_S_2_ and of zidebactam is C_13_H_21_N_5_O_7_S. For the 2D structures, please see Figure S9 of the Supplementary material.

### 3.4. Cefepime/Taniborbactam

Taniborbactam (VNRX-5133) is a novel, boronate derivative, β-lactamase inhibitor that directly inhibits Ambler class A, B, C, and D enzymes. It exhibits promising in vitro activity against OXA-48, KPC, and MBL-CRE isolates [138]. Differential susceptibility profiles of the MBL subclasses to the cefepime/taniborbactam (CEFI/TANI) combination have been described—CEFI/TANI is highly active against VIM-producing *Enterobacterales* [138]. Recent data demonstrate that taniborbactam restores cefepime’s activity against NDM-producing strains [138]. In an in vitro study from Spain, 90% of meropenem-resistant (MIC > 8 mg/L) *Enterobacterales* were susceptible to CEFI/TANI, including KPC-, MBL-, and OXA-48-type producers. CEFI/TANI was the most active agent against OXA-48-type and MBL-producing isolates compared to ceftazidime-avibactam, ceftolozane-tazobactam, IMI/CILA/RELE, and MER/VARB [139]. Notably, taniborbactam remains active against novel KPC-variants, which are resistant to CAZ/AVI. However, the accumulation of mutations can confer CEFI/TANI non-activity against *K. pneumoniae* [140].

Comparing isolates’ susceptibility to CEFI/TANI with that of CEFI/ZIDE demonstrated that 84.1% of KPC- and 75% of MBL-producing isolates had a MIC ≤ 2  mg/L vs. 100% and 96.4%, respectively [138]. CEFI/TANI showed high in vitro activity against VIM-producing *Enterobacterales*, but no significant activity against IMP-producing, while further research is needed to assess activity against NDM-producing stains [117].

Concerning anti-*Pseudomonas* activity, CEFI/TANI had the highest efficacy compared with CAZ/AVI, ceftolozane-tazobactam, IMI/CILA/RELE, and MER/VARB, with 68% of strains being susceptible to CEFI/TANI. In particular, blaGES isolates and MBL-producing strains were the most susceptible isolates [139]. However, although it presented activity against *S. maltophilia*, it failed to do so against *A. baumannii* [141].

#### 3.4.1. Pharmacokinetic-Pharmacodynamic Issues

The pK/pD index best associated with taniborbactam inhibitory activity was fAUC_0–24_/MIC in a neutropenic murine thigh infection model [141].

The dosing regimen of CEFI/TANI suggested for phase 3 trials was 2 g/0.5 g TDS as a 2 h infusion [141]. Cefepime’s and taniborbactam’s pharmacokinetics are similar, and both are primarily excreted in the urine. Dose adjustment is needed for different degrees of renal impairment because increased plasma concentrations of drugs are noted as renal function decreases [142]. In a murine cUTI model, cefepime/taniborbactam was effective against cefepime-resistant *Enterobacterales*, *P. aeruginosa*, and *S. maltophilia* clinical isolates with MICs ≤ 32 mg/L [143].

#### 3.4.2. Clinical Studies

Currently, a phase 3 clinical trial (NCT03840148) comparing CEFI/TANI with meropenem in cUTIs has completed recruitment, and results are pending [144]. The developing company released a press report stating that CEFI/TANI was non-inferior to meropenem, as 70% of participants belonging to the microbiological intent-to-treat population who received the study drug had a clinical and microbiologic response at the test-of-cure visit vs. 58% of the control subjects [112].

In summary, cefepime/zidebactam and cefepime/taniborbactam may constitute significant new weapons in our armamentarium against, at least, cUTIs [120,145] caused by a wide range of MDR-GNB, including infections caused by CRE isolates expressing MBLs and OXA-type enzymes.

#### 3.4.3. Molecular Formula

The molecular formula of cefepime is C_19_H_24_N_6_O_5_S_2_ and of taniborbactam is C_19_H_28_BN_3_O_5_. For the 2D structures, please see Figure S10 of the Supplementary material.

### 3.5. Sulbactam-Durlobactam

Sulbactam is a widely used first-generation β-lactamase inhibitor, which also possesses intrinsic β-lactam activity against *A. baumannii.* Durlobactam (ETX2514) is a novel diazabicyclooctenone β-lactamase inhibitor that effectively inhibits class A, C, and D β-lactamases. Although it lacks anti-MBL activity, it possesses a wide range of anti-D activity against the OXA family of enzymes [146]. The combination of sulbactam and durlobactam (SUL/DUR) exhibits potent inhibition against MDR *Acinetobacter* species. An in vitro study reported that 96.9% of CR-*A. baumannii* isolates were susceptible to SUL/DUR [147], while in another study of 141 (55% colistin-resistant) CR-*A. baumannii* clinical isolates, only 11 (8%) were resistant to SUL/DUR [148]. Regarding the sensitive isolates, the MIC_50_ and MIC_90_ were 0.5 mg/L and 4 mg/L, respectively [148]. Notably, in a Greek in vitro study, SUL/DUR’s activity against CR-*A. baumannii* was enhanced by adding imipenem, which further lowered the MIC_90_ of SUL/DUR two-fold [149].

#### 3.5.1. Pharmacokinetic-Pharmacodynamic Issues

The optimal target attainment against *Acinetobacter* species was an SUL/DUR dosing scheme of 1 g/1 g QDS via a prolonged, 3 h infusion [150]. Meanwhile, SUL/DUR is renally cleared, and dosing should be reduced in patients with severe renal dysfunction, while it can be removed by hemodialysis [151]. Adequate intrapulmonary penetration is achieved, with an ELF to total plasma ratio of 55% for sulbactam and 37% for durlobactam [151].

#### 3.5.2. Clinical Studies

In a phase 2 clinical trial, including 80 patients with cUTIs, SUL/DUR was administered, combined with imipenem-cilastatin, as 1 g/1 g intravenously QDS and was well-tolerated. The most common negative events reported were headache, diarrhea, nausea, and phlebitis, and no serious adverse events occurred. Although no *A. baumannii* infection was detected, the overall rates of success in the microbiologically modified intention-to-treat (m-MITT) population were similar in both groups (SUL/DUR plus imipenem-cilastatin vs. placebo plus imipenem-cilastatin), while in patients with imipenem-resistant pathogens (*n* = 7) response rates were higher than placebo (100% vs. 75%) [152].

A phase 3 clinical trial (NCT03894046) evaluating the efficacy and safety of intravenous SUL/DUR, compared with colistin in HAP, VAP, and bacteremia caused by *A. baumannii-calcoaceticus complex* has completed recruitment, and results were announced in October 2021. All patients had received imipenem/cilastatin as a background antimicrobial, and approximately 95% of *Acinetobacter* isolates were resistant to carbapenem. Sulbactam-durlobactam was non-inferior to colistin, and the 28-day all-cause mortality was 19% and 32.3%, respectively. The combination presented a better clinical cure at the test-of-cure visit and fewer nephrotoxicity events than colistin [153].

In summary, sulbactam-durlobactam is a potential option for treating severe infections caused by carbapenem-resistant *A. baumannii* infections as it retains activity against OXA-enzymes, which are often responsible for that resistance phenotype.

#### 3.5.3. Molecular Formula

The molecular formula of sulbactam is C8H11NO5S and of durlobactam is C8H11N3O6S. For the 2D structures, please see Figure S11 of the Supplementary material.

### 3.6. Sulopenem

Sulopenem is a synthetic thiopenem β-lactam antimicrobial targeting PBP [154]. It has activity against ESBL-producing and AmpC-producing *Enterobacterales* [155]. Additionally, sulopenem exhibits excellent in vitro activity against urinary isolates of *E. coli*, which is not affected by resistance to trimethoprim-sulfamethoxazole and ciprofloxacin [156]. Sulopenem exhibited excellent in vitro activity against *Enterobacterales* isolated from the urine of Canadian patients, with MIC_90_ values ranging from 0.06 to 0.5 mg/L, for all species tested [155]. Additionally, similar to meropenem, it retained its activity against trimethoprim-sulfamethoxazole and ciprofloxacin non-susceptible *E. coli* strains, MDR-*E. coli* (defined as resistant to ≥3 agents from different antimicrobial classes), and AmpC- and ESBL-producing *E. coli* isolates, with MIC values ranging from 0.015 to 0.12 µg/mL [156]. It is not active against *P. aeruginosa*, *S. maltophilia*, and *B. cepacia*.

#### 3.6.1. Pharmacokinetic-Pharmacodynamic Issues

Sulopenem, similarly to other carbapenems, demonstrates time-dependent bacterial kill activity. The optimal fT/MIC varied according to the studied pathogen; for example, a 2-log10 kill of a ESBL-producing *K. pneumoniae* isolate, in an immunocompetent murine thigh infection model, ranged from 12% to 28% [154].

Sulopenem can be administered both intravenously (CP-70,429) and orally as a prodrug, sulopenem etzadroxil (PF-03709270), enabling therapy on an outpatient basis, as well as a step-down therapy from intravenous (IV) to oral administration. Oral bioavailability ranges from 20.1% to 33.6% and improves substantially when the drug is co-administered with food and probenecid. Concentrations achieved in urine vary, as approximately 35.5% of the 1000 mg dose was recovered in urine [154].

Sulopenem etzadroxil 500 mg/probenecid g is administered twice daily as a bilayer tablet, while IV sulopenem is given as 1000 mg once daily and reduced to 250 mg once daily in patients with CrCl < 30 mL/min [157]. Like most β-lactams, a high percentage of time free concentrations that is above the MIC (%fT > MIC) is considered the desired pD target [154], and as a result, sulopenem 1 g IV was infused over 3 h in a phase 3 clinical trial [157].

#### 3.6.2. Clinical Studies

Sulopenem’s role in treating uncomplicated and complicated UTIs, and complicated intra-abdominal infections has been studied in phase 3 clinical trials. The authors of the first trial compared IV sulopenem followed by oral sulopenem to IV ertapenem followed by oral ciprofloxacin or amoxicillin-clavulanate for cUTIs. Sulopenem was not non-inferior to the comparator regimen regarding the overall clinical and microbiological response (67.8% vs. 73.9%). It exhibited high clinical success rates (89.4% for sulopenem, and 88.4% for ertapenem). However, microbiological success was lower than ertapenem (71.2% vs. 78.0%), as asymptomatic bacteriuria after completion of therapy was a more common finding in the study group, mainly due to better eradication of quinolone-susceptible pathogens with oral ciprofloxacin following IV ertapenem. In patients with ciprofloxacin-resistant isolates, overall success and asymptomatic bacteriuria rates were similar between the two groups. The most common adverse events reported were headache and diarrhea [157].

In uncomplicated UTIs, overall, oral sulopenem etzadroxil/probenecid was non-inferior, though it was superior to ciprofloxacin when treating ciprofloxacin-resistant pathogens [157]. However, in July 2021, the FDA denied a New Drug Application for sulopenem etzadroxil/probenecid for treating adult women with uncomplicated UTIs due to quinolone non-susceptible uropathogens, and additional data were requested to support the approval. A new phase 3 clinical trial (REnewed ASsessment of Sulopenem in uncomplicated UTI caused by Resistant *Enterobacterales* (REASSURE), NCT05584657) has started recruiting patients for the evaluation of oral sulopenem compared to amoxicillin/clavulanate for treating adult women with uncomplicated UTI [158]. Upon its completion, the company plans to resubmit a New Drug Application to the FDA in 2024.

Concerning cIAIs, according to the results announced from the SURE 3 clinical trial (Sulopenem for Resistant Enterobacteriaceae (SURE) 3 trial) on December 2019, sulopenem missed the primary endpoint and its non-inferiority to ertapenem was not established [159]. In summary, sulopenem may have a role in the inpatient and step-down therapy of cUTIs caused by ESBL, AmpC *Enterobacterales*, and quinolone-resistant isolates.

#### 3.6.3. Molecular Formula

The molecular formula of sulopenem is C12H15NO5S3. For the 2D structure of sulopenem, please see Figure S12 of the Supplementary material.

### 3.7. Tebipenem

Tebipenem/pivoxil hydrobromide (SPR994) is an orally bioavailable carbapenem prodrug. Tebipenem is the active form with activity against *Enterobacterales* pathogens, including MDR strains [160]. It is less active against *P. aeruginosa* and inactive against KPC- and MBL-producing *Enterobacterales*. Specifically, tebipenem’s MIC_50_ was ≤0.06 μg/mL for ESBL and AmpC-producing isolates of *E. coli* and *K. pneumoniae*, while it was >2 μg/mL for *P. aeruginosa* [161].

#### 3.7.1. Pharmacokinetic-Pharmacodynamic Issues

It is administered orally with a dose of 600 mg TDS, achieving bioavailability of 50–60%, and it shows time-dependent bactericidal efficacy [162,163]. It is primarily excreted through the kidneys, achieving high urine concentration. Around 38–64% of the administered dose (600 mg) is recovered from urine, and plasma concentrations increase significantly with varying degrees of renal impairment, requiring a dose adjustment for creatinine clearance below 50 mL/min [164]. Efficient intrapulmonary penetration of tebipenem in healthy subjects was reported in a phase 1 trial (NCT04710407) [162], raising the question of whether the drug can be used in lower tract respiratory infections caused by MDR strains.

#### 3.7.2. Clinical Studies

ADAPT-PO, a phase 3 clinical trial, has been recently completed. According to the results, oral tebipenem pivoxil hydrobromide was non-inferior to IV ertapenem for treating patients with cUTIs and acute pyelonephritis concerning the primary endpoint of overall response at the test-of-cure visit (58.8% and 61.6% of the patients, respectively). The overall response at the end-of-treatment visit was 97.3% in the tebipenem pivoxil hydrobromide group and 94.5% in the ertapenem group. The development of asymptomatic bacteriuria was the main reason for the decrease in response rates from the test-of-cure to the end-of-treatment visit. Additionally, the efficacy was similar in both groups regarding fluoroquinolone-resistant, ESBL-producing, and trimethoprim-sulfamethoxazole-resistant strains [165].

However, in June 2022, FDA rejected Spero Therapeutics’ application for approval of tebipenem for outpatient treatment of cUTIs, requesting further investigations, and subsequently, the company suspended commercialization activities.

In summary, with its oral formulation, tebipenem may be a therapeutic option in patients with community-acquired UTIs caused by ESBL and AmpC-producing *Enterobacterales*, as well as quinolone- and trimethoprim/sulfamethoxazole-resistant strains.

#### 3.7.3. Molecular Formula

The molecular formula of tebipenem is C16H21N3O4S2. For the 2D structure of tebipenem, please see Figure S13 of the Supplementary material.

### 3.8. Benapenem

Benapenem is a novel carbapenem with activity against ESBL-producing *Enterobacterales*. It exhibited in vitro activity against ESBL-producing *E. coli*, *K. pneumoniae*, and *Enterobacter cloacae* strains with an MIC_90_ ≤ 0.5 μg/mL [166].

#### 3.8.1. Pharmacokinetic-Pharmacodynamic Issues

Its bacteriostatic and bactericidal effect depends on the percentage of time that the free drug concentration remains above the MIC (%fT > MIC). A target of %fT > MIC above 60% is proposed to ensure bactericidal efficacy [166]. According to results from phase 1 trials, benapenem has a long half-life period, similar to ertapenem, can be administered as a 1000 mg OD dose, and is well tolerated [167]. In a pD study evaluating benapenem, administration in patients with mild to moderate renal impairment, the authors recommended no dose adjustment in mild to moderate renal impairment, as plasma concentrations did not increase significantly in patients with renal dysfunction [168].

#### 3.8.2. Clinical Studies

A phase 2/3 clinical trial (NCT04505683) has completed recruitment to evaluate the efficacy and safety of benapenem in cUTIs. Recently, Sihuan Pharmaceutical has licensed out the rights of benapenem to New Asia Pharmaceutical [169].

#### 3.8.3. Molecular Formula

The molecular formula of benapenem is C22H28N4O7S2. For the 2D structure of benapenem, please see Figure S14 of the Supplementary material.

## 4. Discussion

Critically ill patients with sepsis require prompt and appropriate empirical antimicrobial treatment. If the patient has recently received carbapenem treatment or is colonized with CR-pathogens, and a rational initial regimen is the combination of two non-beta lactam drugs, such as colistin, aminoglycoside, tigecycline, or fosfomycin, we must cover for potential CRE and non-fermenter pathogens [170]. Alternatively, when we have evidence for a CRE infection, but we do not know the resistance phenotype, we can initially treat with a novel beta-lactam/beta-lactamase inhibitor plus aztreonam. When the susceptibility test results become available, we can de-escalate treatment accordingly [171].

According to ESCMID recommendations for targeted treatment of GNBs, monotherapy with MER/VARB, or CEFID in patients with susceptible bacteria is strongly encouraged, though based on insufficient evidence [28]. Monotherapy is possibly the best approach to treat KPC-producing *Enterobacterales*, as MER/VARB, CEFID, and IMI/CILA/RELE present over 90% in vitro activity against these isolates. Thus, choosing the best anti-KPC active drug can be challenging. MER/VARB showed in vitro possible reduced potential for resistance development compared to CAZ/AVI [172]. Meanwhile, many KPC-mutants that developed increased MIC to CAZ/AVI also presented increased MICs to CEFID [5]. KPC mutations can develop resistance to IMI/CILA/RELE, and cross-resistance to MER/VARB [173]. However, KPC is not the only mechanism for CR. Unfortunately, many beta-lactamase-producing microorganisms can produce up to four beta-lactamases and carry determinants for many antimicrobial classes beyond beta-lactams [92,174]. Nonetheless, the aminoglycosides, polymyxins, and high-dose tigecycline could increase coverage if combined with the beta-lactams [175,176].

For the OXA-48-like-producing *Enterobacterales,* CEFID can be used. Notably, there has recently been a shift from KPC- to MBL-carbapenemases after the launch of modern, KPC-active, beta-lactam/beta-lactamase inhibitors [177]. MBL-production often added onto other class beta-lactamase-production, limits the novel treatment options to just two: CEFID and CAZ/AVI plus aztreonam. Moreover, there is scarce in vitro data on the synergistic potential of combining IMI/CILA/RELE with aztreonam. Plazomicin remains an option for treating MBL-associated cUTIs or can be used compassionately if available. Finally, we are eagerly waiting the launch of AZT/AVI, CEFI/ZIDE, and CEFI/TANI for treating MBL-CRE infections.

*A. baumannii*, despite the in vitro susceptibility, proved resilient to CEFID, as more CEFID-treated patients died than those who received the comparator, which is the best available treatment. Novel tetracycline class drugs comprise ERA and OMA. ERA has been successfully studied for treating cIAIs, although real-world data provide limited data for its use against CR-*A. baumannii* pneumonia. OMA is a more intriguing prospect: it presents higher levels in the blood and lung compartments than other modern tetracycline antimicrobials. It might be used for treating *A. baumannii* if combined with other available drugs. However, it has not been tested yet. Meanwhile, SUL/DURL presents anti-OXA activity and can be a valuable treatment option when available.

CEFID and IMI/CILA/RELE may cover even CAZ/AVI- and ceftolozane-resistant *P. aeruginosa* isolates and are conditionally recommended for CRPA infections [28].

Meanwhile, the treating physicians should be vigilant about resistance in vivo and repeat susceptibility testing as resistance to the novel drugs may evolve through various mechanisms [178].

A systems-based, machine learning approach revealed: (i) a plasmid carrying multiple resistance genes; (ii) the beta-lactam concentration yielding 50% of maximum killing; and (iii) hypotension ranked most important for 30-day mortality. The combination of drugs might have benefited the patients by enhancing bacterial killing and decreasing bacterial regrowth [179]. Moreover, little is known about the bacterial inoculum effect on these novel drugs’ therapeutic efficacy [180]. At the usual inocula of 10^5^ CFU/mL, many bacteria were susceptible to MER/VARB (70%) and CEFID (85%), and IMI/CILA/RELE (45%). At a higher inocula of 10^7^, the susceptibility got worse at 50%, 12%, and 28%. All the above may indicate that in severe infections failing to improve rapidly, adding a second drug might benefit the patient outcomes.

In summary, CEFID presents the widest in vitro Gram-negative spectrum that includes CRE, CRPA, MBLs and CRAB. However, clinical data showed a numerically increased mortality in CEFID’s arm compared to BAT for nosocomial pneumonia and BSI/sepsis, and further clinical research is needed to establish its clinical efficacy, especially for MBL-producing and CRAB. The above mentioned carbapenemases classes B (MBLs) and D (OXA), and CRAB are not included in the spectrum of IMI/CILA/RELE and MER/VARB, which retain stability to carbapenemases classes A and C. CEFID and IMI/CILA/RELE cover more *P. aeruginosa* strains than MER/VARB. Notably, although relebactam enhances imipenem action, vaborbactam cannot improve meropenem’s anti-*Pseudomonas* activity. ERA targets an extensive spectrum of intestinal bacteria plus *A. baumannii.* It was clinically studied and licensed for treating cIAIs which revealed that it has poor pK in the urinary tract and is not indicated for cUTIs. OMA use against *A. baumannii*, is not warranted, and despite its in vitro activity, its efficacy in critically ill patients remains to be studied. PLAZ is indicated for cUTIs in patients with no or limited alternative options, and it is more active against *Enterobacterales*, including CREs, than non-lactose-fermenters (inactive in vitro against *A. baumannii* and *S.maltophilia* and variable activity against *P. aeruginosa*).

Among the phase 3 antimicrobials, AZT/AVI, CEFI/ZIDE, and CEFI/TANI can target several MBL-producing CREs, while SUL/DUR presents the sole antibacterial agent with promising activity against *A. baumannii*. CEFI/ENM displays excellent anti-ESBL activity coupled with adequate lung penetration. Finally, sulopenem, tebipenem, and biapenem are novel carbapenems targeting ESBL-producing *Enterobacterales*, without anti-*Pseudomonas* activity; notably, sulopenem and tebipenem are the first-class members that can also be administered orally.

Detailed comparison of the novel branded antibiotics and the antibiotics in phase 3, are presented in Table 1 and Table 2, respectively.

The highlights of the manuscripts are presented in Appendix A.

## 5. Conclusions

The number of new antimicrobial agents are not keeping up pace with antimicrobial resistance development and patients’ needs. Their vast majority represent modifications of existing chemical structures, addressing specific mechanisms of resistance, rather than new drug classes with unique mechanisms of actions.

Most of the recently approved agents and those in late-stage clinical development can withstand the inactivation by serine-beta-lactamase enzymes. However, the global spread of metallo-beta-lactamase-producing GNBs and of carbapenemases-producing *A. baumannii* remain the hardest challenges to address and represent an unmet clinical need, as very few of these novel agents possess in vitro activity and potential clinical effectiveness against these difficult-to-treat pathogens.

## Figures and Tables

**Table 1:** Characteristics of novel approved anti-Gram-negative antimicrobial agents.

Drug	Drug Class	Bacterial Spectrum	Drug Stable to Beta-Lactamase Type					Approval Year/Indication in Adults		Recommendations		Route//Dosage
			KPC	MBL	AmpC	OXA	ESBL	FDA	EMA	IDSA	ESCMID	
**Cefiderocol**	BL	GNB; not GPB and anaerobes	yes	yes	yes	yes	yes	2019/cUTI, HAP/VAP	2020/aerobic GNB when limited treatment options	CRE: cUTI	CRE ^#^, CRAB, CRPA *	IV//2 g TDS (2 g QDS if augmented renal clearance)
**Meropenem/** **vaborbactam**	CP/BLI	GNB, GPB, anaerobes; not MRSA, VRE; VARB cannot enhance activity against *A. baumannii* and *Pseudomonas aeruginosa*	yes §	no	yes	no	yes	2017/cUTI	2018/cUTI, cIAI, HAP/VAP, when limited options	CRE: cUTI, infections elsewhere	CRE	IV//4 g TDS
**Imipenem/** **cilastatin/** **relebactam**	CP/ BLI	GNB, GPB, anaerobes; not MRSA, VRE; RELE enhances activity against *P. aeruginosa*, contrary to *A. baumannii*; not *Morganellaceae*	yes	no	yes	no	yes	2019/cUTI and cIAI with limited options, HAP, VAP	2021/HAP/VAP, BSI possibly secondary to pneumonia, when limited options	CRE: cUTI, infections elsewhere CRPA: cUTI	CRPA *	IV//1.25 g QDS
**Eravacycline**	TC	GNB, GPB, anaerobes; MRSA, VRE active; possibly against *A. baumannii*; not *P. aeruginosa*; less active against *Morganellaceae*	-	-	-	-	-	2018/cIAI	2018/cIAI	-	CRAB: No data	IV//1 mg/kg BD
**Omadacycline**	TC	GNB, atypical, GPB, anaerobes; MRSA, VRE active; may display activity against *A. baumannii*; not against *P.aeruginosa* and *Morganellaceae*	-	-	-	-	-	2018/CAP, ABSSSI	-	-	CRAB: No data	IV//1st day 200 mg, later 100 mg OD; oral//450 mg for 2 days followed by 300 mg
**Plazomicin**	AG	Aerobic GNB, ESBL-E, CRE (including MBL); stable against some AG-resistant *Enterobacterales*	-	-	-	-	-	2018/cUTI	No	-	CRE: cUTI	IV//15 mg/Kg/day

There can be some exceptions to the rule ^#^ Inconclusive evidence, conditional. recommendation * Insufficient evidence § Low resistance potential of some KPC variants during treatment; **Abbreviations:** ABSSSI—acute bacterial skin and skin structure infection; AG—aminoglycoside; AmpC—chromosomally encoded beta-lactamase; BL—beta-lactam; BLI—beta-lactamase inhibitor; cIAI—complicated intraabdominal infection; CAP—community-acquired pneumonia; CRAB—carbapenem-resistant *Acinetobacter baumannii*; CP—carbapenem; CRE—carbapenem-resistant Enterobacterales; CRPA—carbapenem-resistant *Pseudomonas aeruginosa*; cUTI—complicated urinary tract infection; EMA—European Medicines Agency; ERA—eravacycline; ESBL—extended spectrum beta-lactamases; ESBL-E—extended spectrum beta-lactamase-producing Enterobacterales; FDA—Food and Drug Administration; GNB—Gram-positive bacteria; GPB—Gram-positive bacteria; HAP—hospital-acquired pneumonia; IMP/CILA/RELE—imipenem/cilastatin/relebactam; IV—intravenous; KPC—*Klebsiella pneumoniae* carbapenemase; MBL—metallo-β-lactamase; MDR—multidrug-resistant; MER/VARB—meropenem/vaborbactam; MRSA—methicillin-resistant *Staphylococcus aureus*; NDM—New Delhi metallo-β-lactamase; OXA—oxacillinase; OMA—omadacycline; PLAZ—plazomicin; OD—once daily; QDS—four times daily; rRNA—ribosomal ribonucleic acid; SD—standard deviation; TC—tetracycline; TDS—thrice daily; VAP—ventilator-acquired pneumonia); VIM—Verona integron-encoded metallo-β-lactamase; VRE—vancomycin-resistant *Enterococcus*.

**Table 2:** Characteristics of novel anti-Gram-negative antimicrobial agents in phase 3 clinical trials.

Drug	Drug Class	Bacterial Pectrum	Drug Stable to Beta-Lactamase Type					Potential Indications	Ongoing Trials (Phase 3)	Route//Dosage	Comment
			KPC	MBL	AmpC	OXA	ESBL				
**Aztreonam/avibactam**	MB/BLI	GNB; less effective against MBL-producing *P. aeruginosa*	yes	yes	yes	yes	yes	cIAI, HAP, VAP, cUTI, BSI	NCT03580044	IV//500/167 mg loading dose, 1500/500 mg QDS	Combination that covers both serine and MBL carbapenemases
**Cefepime/** **enmetazobactam**	BL/BLI	GNB, GPB; ineffective against *A. baumannii*, CRE, and MRSA	no	no	yes/no	no	yes	cUTI, HAP, VAP	NCT03687255	IV//2 g/500 mg TDS	Excellent class A, anti-ESBL activity; Potential carbapenem-sparing drug; Penetrates ELF easily
**Cefepime/** **zidebactam**	BL/BLI	GPB (no MRSA); GNB (not *A. baumannii*)	yes	yes	yes	yes	yes	cUTI	NCT04979806	IV//2 g/1 g TDS	Phase 3 study recruiting patients
**Cefepime/** **taniborbactam**	BL/BLI	GPB (not MRSA), GNB, (not *A. baumannii*)	yes	yes	yes	yes	yes	cUTI	NCT03840148	IV//2 g/0.5 g TDS	Preliminary results from phase 3 cUTI study: comparable efficacy to MER
**Sulbactam/** **durlobactam**	BL/BLI	GNB, particularly *Acinetobacter* spp.; active against *Burkholderia cepacia*; not *P. aeruginosa*	yes	no	yes	yes	yes	*Acinetobacter* infections	NCT03894046	1 g/1 g QDS	Phase 3, pneumonia and BSI study. Better clinical cure and less nephrotoxicity than colistin (preliminary results)
**Sulopenem**	CP	GPB (not MRSA); GNB (not *P.aeruginosa*, *Stenotrophomonas maltophilia*, *B. cepacia)*	no	no	yes	no	yes	cUTI, uUTI, cIAI	NCT03357614, NCT03358576	Oral//sulopenem etzadroxil 500 mg/probenecid 500 mg BD; IV//1000 mg OD	Phase 3 trial of uncomplicated UTI, recruiting patients
**Tebipenem**	CP	GPB (not MRSA); GNB (not *P.aeruginosa*, *A. baumannii*)	no	no	yes	no	yes	cUTI	NCT03788967	Oral//600 mg TDS	Excellent anti-ESBL activity
**Benapenem**	CP	Like other CPs	no	no	yes	no	yes	cUTI	NCT04505683	IV//1000 mg OD	Excellent anti-ESBL activity

**Abbreviations:** AmpC—chromosomally encoded beta-lactamase; BD—twice daily; BL—beta-lactam; BLI—beta-lactamase inhibitor; BSI—bloodstream infection; CP—carbapenem; CR—Carbapenem resistant; CRE—Carbapenem-resistant Enterobacteriaceae; cIAI—complicated intraabdominal infections; cUTI—complicated urinary tract infections; ELF—epithelial lining fluid; ESBL—Extended Spectrum Beta-Lactamase; GNB—Gram-positive bacteria; GPB—Gram-positive bacteria; HAP—hospital acquired pneumonia; KPC—*Klebsiella pneumoniae* carbapenemase; MB—monobactam; MER—meropenem; MBL—metallo-β-lactamases; MDR—Multidrug-resistant; OD—once daily; OXA—oxacillinase; QDS—four times daily; TDS—thrice daily; VAP—ventilator associated pneumonia.

## Data Availability

Not applicable.

## Supplementary Materials

The following supporting information can be downloaded at: https://www.mdpi.com/article/10.3390/antibiotics12040761/s1, Figure S1: Cefiderocol; Figure S2: Imipenem-Cilastatin-Relebactam; Figure S3: Meropenem—Vaborbactam; Figure S4: Eravacycline; Figure S5: Omadacycline; Figure S6: Plazomicin; Figure S7: Aztreonam—Avibactam; Figure S8: Cefepime—Enmetazobactam; Figure S9: Cefepime—Zidebactam; Figure S10: Cefepime—Taniborbactam; Figure S11: Sulbactam—Durlobactam; Figure S12: Sulopenem; Figure S13: Tebipenem; Figure S14: Benapenem. References [181,182] are cited in the supplementary materials.

## Abbreviations

ABSSSIs: Acute Bacterial Skin and Skin Structures Infections; AG: aminoglycoside; AMEs: aminoglycosides-modifying enzymes; AMR: antimicrobial resistance; APACHE II: Acute Physiology and Chronic Evaluation II; AZT/AVI: aztreonam/avibactam; BAT: best available treatment; BSI: bloodstream infections; CAZ/AVI: ceftazidime-avibactam; CABP: community-acquired bacterial pneumonia; CEFI/ENM, cefipime/enmetazobactam; CEFI/TANI, cefipime/taniborbactam; CEFI/ZIDE, cefipime/zidebactam; CEFID: cefiderocol; CFU: colony-forming unit; cIAI: complicated intraabdominal infection; COLI: colistin; CR: carbapenemase-resistance; CRPA: carbapenemase-resistant *Pseudomonas aeruginosa*; CRE: carbapenemase-resistant *Enterobacterales*; CTX-M, cefotaxime-hydrolyzing-Munich; cUTI: complicated urinary tract infection; ELF: epithelial lining fluid; EC: alveolar cells; ERA: eravacycline; ESBL: extended-spectrum beta-lactamases; ESCMID: European Society of Clinical Microbiology and Infectious Diseases; EUCAST: European Committee on Antimicrobial Susceptibility Testing; fAUC/MIC: 24 h area under the concentration time curve for the drug to microbe’s minimal inhibitory concentration ratio; fT/MIC: total time the drug concentration is above the minimal inhibitory concentration for the microbe to microbe’s minimal inhibitory concentration ratio; fCmin/MIC: the ratio of the total time the trough drug concentration is above the minimal inhibitory concentration to microbe’s minimal inhibitory concentration; %fT > MIC: the percentage of time that the drug concentration is above the microbe’s minimal inhibitory concentration; GNB: Gram-negative bacteria; HAP: hospital-acquired pneumonia; HCAP: healthcare-associated pneumonia; ICU: intensive care unit; IMI/CILA/RELE: imipenem/cilastatin/relebactam; IMP: active-on-imipenem; KPC: *Klebsiella pneumoniae,* carbapenemase-producing; MBL: metallo-beta-lactamases; MDR: multidrug-resistant; MER/VARB: meropenem/vaborbactam; MIC: minimal inhibitory concentration; NCP: non-carbapenemase producers; NDM: New Delhi metallo-β-lactamase; OMA: omadacycline; OprD: outer membrane porin D; OXA: oxacillinase; PER-1: Pseudomonas extended resistant; pK/pD: pharmacokinetic/pharmacodynamic; PLAZ: plazomicin; PTA: probability of target attainment; QDS: four times daily; rRNA: ribosomal ribonucleic acid; SUL/DUR: sulbactam-durlobactam; SOFA: sequential organ failure assessment; TDS: three times daily; VIM: Verona integron-mediated; VAP: ventilator-associated pneumonia; XDR, extensively drug-resistant.

## Appendix A

**Highlights—Novel antimicrobial agents (branded during the last 5 years)**

- Cefiderocol is a new siderophore cephalosporin approved for cUTIs and HAP/VAP (FDA/EMA), active against lactose-fermenting and non-fermenting GNBs, including ESBL and carbapenemases-producing strains (including OXA-type serine beta-lactamases and MBLs, although further data are needed regarding clinical activity against MBLs).
- Meropenem/vaborbactam is a beta-lactam/beta-lactamases inhibitor combination approved for cUTIs and cIAIs (FDA), HAP/VAP, associated bacteremia and infections due to GNBs with limited treatment options (EMA). It is active against serine-beta-lactamases of class A (including ESBLs and KPCs) and class C (including AmpC) but is inactive against OXA-type beta-lactamases and MBLs.
- Imipenem/cilastatin/relebactam is a triple combination of a beta-lactam with beta-lactamases inhibitors, approved for cUTIs and cIAIs (FDA), HAP/VAP, associated bacteremia and GNBs with limited treatment options (EMA). It is active against lactose-fermenting and non-fermenting GNBs, including strains producing ESBL/KPC/AmpC (including imipenem-R); it is inactive against *A. baumannii*, and MBLs- producing GNBs.
- Eravacycline is a new glycylcycline approved for cIAIs (FDA/EMA), with a wide spectrum of activity against Gram-positive and Gram-negative pathogens, including ESBL-producing *Enterobacterales* (and some CRE strains) and *A. baumannii* (including some CRAB strains). It is inactive against *P. aeruginosa* and *B. cenocepacia.*
- Omadacycline is a novel tetracycline, approved for ABSSI and CABP (FDA), with a broad spectrum of activity against Gram-positive, Gram-negative and atypical bacteria, including ESBL-producing Enterobacterales and some CRE, and *A.baumannii* (including some CRAB), while it is inactive against *P. aeruginosa*, *P. mirabilis*, *Providencia* spp., and *M. morgannii*. Its role for ICU patients and against MDR-GNBs, such as CRE/CRAB is not yet established. Omadacycline also has an oral formulation.
- Plazomicin is a new semi-synthetic aminoglycoside approved for cUTIs (FDA), active against GNBs (better activity against lactose-fermenting ones), including CREs and AG-resistant producing several AMEs. It is inactive against *A. baumannii* and *S. maltophilia*, while its activity against *P. aeruginosa* is variable.
- Application of stringent antimicrobial stewardship programs, along with pK/pD optimization, may preclude indiscriminate use of these valuable additions to our antimicrobial armamentarium and prolong their self-lives.

**Highlights—Antimicrobials in phase 3 clinical trials**

- Aztreonam-avibactam can be a valuable option for the management of MBL-producing *Enterobacterales*.
- Cefepime-enmetazobactam possesses excellent activity against ESBL-producing Enterobacterales and can be considered as a carbapenem-sparing regimen in cUTIs, and possibly in VAP/HAP as it exhibits good ELF penetration. MBLs are not inhibited by cefepime-enmetazobactam.
- Cefepime-zidebactam and cefepime-taniborbactam are active against a wide range of β-lactamases from classes A, B, C, and D, conferring them a valuable option against MDR-GNB infections.
- Sulbactam-durlobactam’s strong anti-D activity against the OXA family of enzymes, makes it a potential option for carbapenem-resistant *A. baumannii* infections.
- Sulopenem (IV and oral) and tebipenem (oral) may have a role in the treatment of complicated and uncomplicated UTIs caused by ESBL, AmpC *Enterobacterales*, and quinolone-resistant uropathogens.
- Benapenem is another novel carbapenem, currently in phase 2/3 study for cUTIs.
