# Supplementary material for: Novel Antimicrobial Agents for Gram-Negative Pathogens
Source: Antibiotics (Basel). 2023 Apr 16;12(4):761. doi: 10.3390/antibiotics12040761 (PMC10135111; doi:10.3390/antibiotics12040761)
Supplement: Supplementary file 1 [file antibiotics-12-00761-s001.zip › antibiotics-2282177-supplementary.pdf]

SUPPLEMENT

References:

National Center for Biotechnology Information. The PubChem Project. Available from: https://pubchem.ncbi.nlm.nih.gov/ *(accessed 24.03.23)* [Reference 181]

Kim S, Chen J, Cheng T, et al. PubChem 2023 update. *Nucleic Acids Res.* 2023;51(D1):D1373–D1380. doi:10.1093/nar/gkac956 [Reference 182]

S-1. Cefiderocol

Chemical name: Cefiderocol

Pubchem CID: 77843966

Molecular formula: $C_{30}H_{34}C_{l}N_{7}O_{10}S_{2}$

Molecular Weight: 752.2 g/mol

IUPAC Name: (6R,7R)-7-[[(2Z)-2-(2-amino-1,3-thiazol-4-yl)-2-(2-carboxypropan-2-yloxyimino)acetyl]amino]-3-[[1-[2-[(2-chloro-3,4-dihydroxybenzoyl)amino]ethyl]pyrrolidin-1-ium-1-yl]methyl]-8-oxo-5-thia-1-azabicyclo[4.2.0]oct-2-ene-2-carboxylate

2D structure:

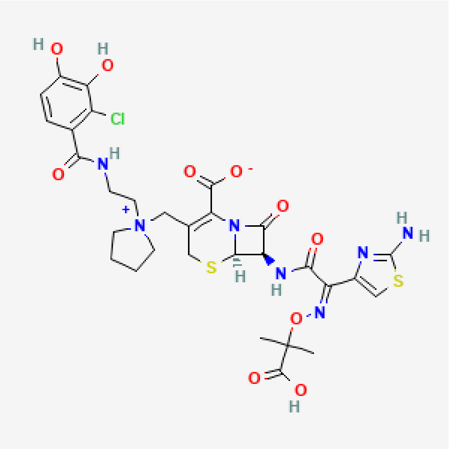

https://pubchem.ncbi.nlm.nih.gov/compound/77843966 *(accessed 24.03.23)*

###

S-2. Imipenem-Cilastatin-Relebactam

Imipenem

Chemical name: Imipenem

Pubchem CID: 104838

Molecular formula: $C_{12}H_{17}N_{3}O_{4}S$

Molecular Weight: 299.35g/mol

IUPAC Name: (5R,6S)-3-[2-(aminomethylideneamino)ethylsulfanyl]-6-[(1R)-1-hydroxyethyl]-7-oxo-1-azabicyclo[3.2.0]hept-2-ene-2-carboxylic acid
2D structure:

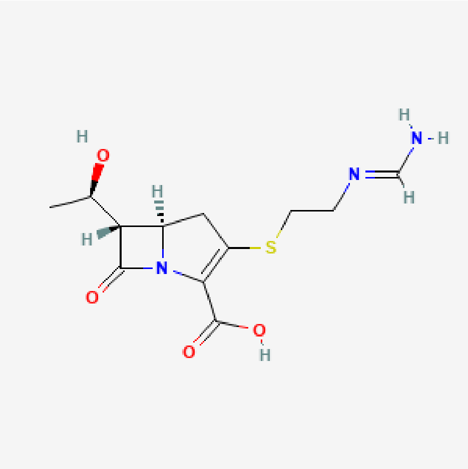

https://pubchem.ncbi.nlm.nih.gov/compound/104838 *(accessed 24.03.23)*

Cilastatin

Chemical name: Cilastatin
Pubchem CID: 6435415
Molecular formula: $C_{16}H_{26}N_2O_5S$
Molecular Weight: 358.5g/mol
IUPAC Name: (Z)-7-[(2R)-2-amino-2-carboxyethyl]sulfanyl-2-[[(1S)-2,2-dimethylcyclopropanecarbonyl]amino]hept-2-enoic acid
2D structure:

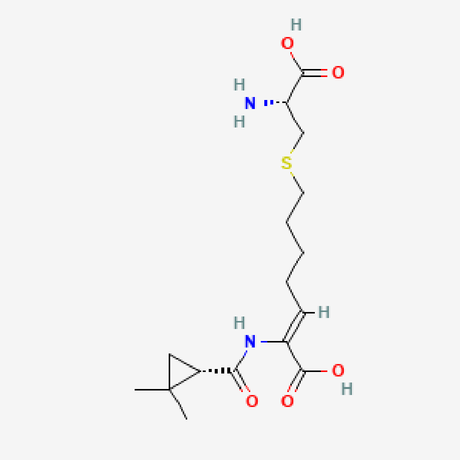

https://pubchem.ncbi.nlm.nih.gov/compound/6435415 *(accessed 24.03.23)*

Relebactam

Chemical name: Relebactam
Pubchem CID: 44129647
Molecular formula: $C_{12}H_{20}N_4O_6S$
Molecular Weight: 348.38g/mol

IUPAC Name: [(2S,5R)-7-oxo-2-(piperidin-4-ylcarbamoyl)-1,6-diazabicyclo[3.2.1]octan-6-yl] hydrogen sulfate
2D structure:

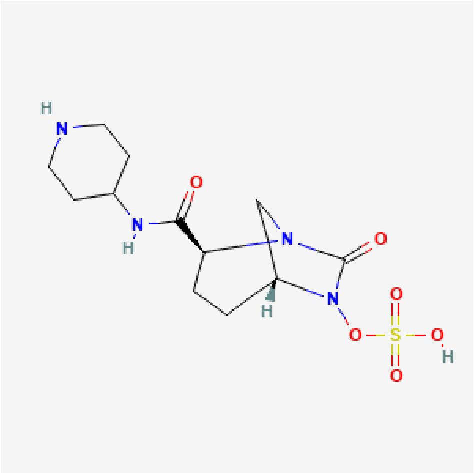

https://pubchem.ncbi.nlm.nih.gov/compound/44129647 *(accessed 24.03.23)*

###

S-3. Meropenem - Vaborbactam

Meropenem

Chemical name: Meropenem
Pubchem CID: 441130
Molecular formula: $C_{17}H_{25}N_3O_5S$
Molecular Weight: 383.5g/mol
IUPAC Name: (4R,5S,6S)-3-[(3S,5S)-5-(dimethylcarbamoyl)pyrrolidin-3-yl]sulfanyl-6-[(1R)-1-hydroxyethyl]-4-methyl-7-oxo-1-azabicyclo[3.2.0]hept-2-ene-2-carboxylic acid

2D structure:

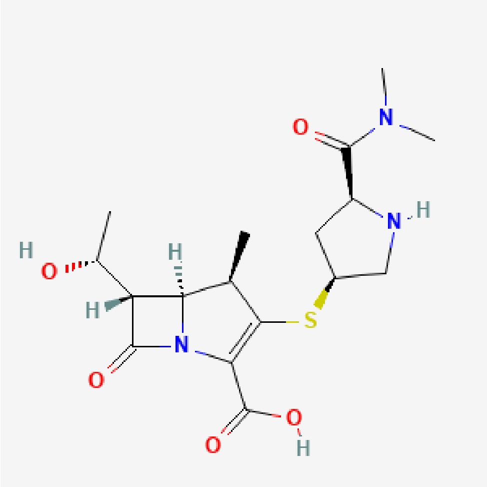

https://pubchem.ncbi.nlm.nih.gov/compound/441130 *(accessed 24.03.23)*

Vaborbactam

Chemical name: Vaborbactam
Pubchem CID: 56649692
Molecular formula: $C_{12}H_{16}BNO_5S$
Molecular Weight: 297.14g/mol
IUPAC Name: 2-[(3R,6S)-2-hydroxy-3-[(2-thiophen-2-ylacetyl)amino]oxaborinan-6-yl]acetic acid
2D structure:

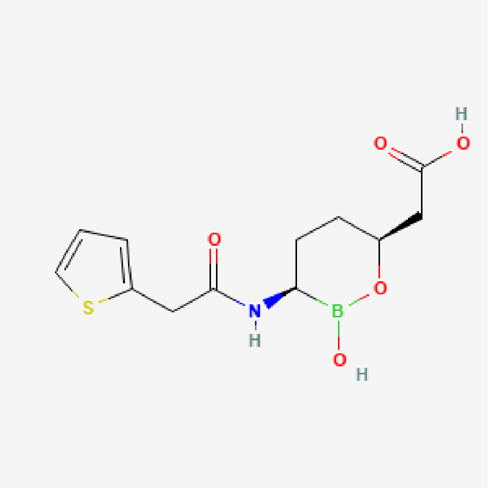

https://pubchem.ncbi.nlm.nih.gov/compound/56649692 *(accessed 24.03.23)*

###

S-4. Eravacycline

Chemical name: Eravacycline
Pubchem CID: 54726192
Molecular formula: $C_{27}H_{31}FN_4O_8$
Molecular Weight: 558.6g/mol
IUPAC Name: (4S,4aS,5aR,12aR)-4-(dimethylamino)-7-fluoro-1,10,11,12a-tetrahydroxy-3,12-dioxo-9-[(2-pyrrolidin-1-ylacetyl)amino]-4a,5,5a,6-tetrahydro-4H-tetracene-2-carboxamide
2D structure:

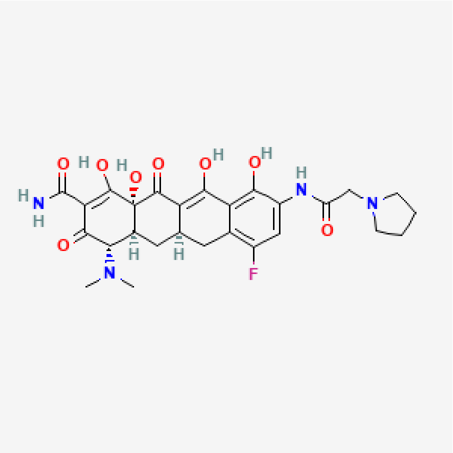

https://pubchem.ncbi.nlm.nih.gov/compound/56951485 *(accessed 24.03.23)*

###

S-5. Omadacycline

Chemical name: Omadacycline
Pubchem CID: 54697325
Molecular formula: $C_{29}H_{40}N_4O_7$
Molecular Weight: 556.6g/mol
IUPAC Name: (4S,4aS,5aR,12aR)-4,7-bis(dimethylamino)-9-[(2,2-dimethylpropylamino)methyl]-1,10,11,12a-tetrahydroxy-3,12-dioxo-4a,5,5a,6-tetrahydro-4H-tetracene-2-carboxamide
2D structure:

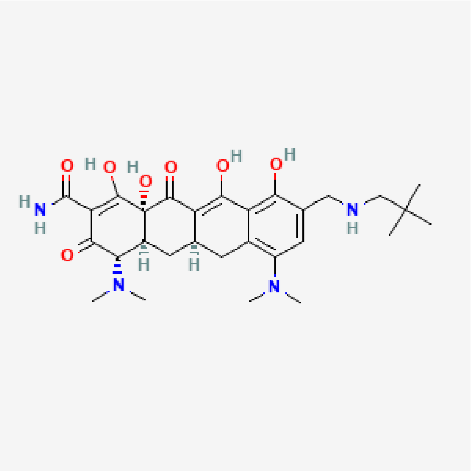

https://pubchem.ncbi.nlm.nih.gov/compound/54697325 *(accessed 24.03.23)*

###

S-6. Plazomicin

Chemical name: Plazomicin
Pubchem CID: 42613186
Molecular formula: $C_{25}H_{48}N_6O_{10}$

Molecular Weight: 592.7g/mol
IUPAC Name: (2S)-4-amino-N-[(1R,2S,3S,4R,5S)-5-amino-4-[[(2S,3R)-3-amino-6-[(2-hydroxyethylamino)methyl]-3,4-dihydro-2H-pyran-2-yl]oxy]-2-[(2R,3R,4R,5R)-3,5-dihydroxy-5-methyl-4-(methylamino)oxan-2-yl]oxy-3-hydroxycyclohexyl]-2-hydroxybutanamide
2D structure:

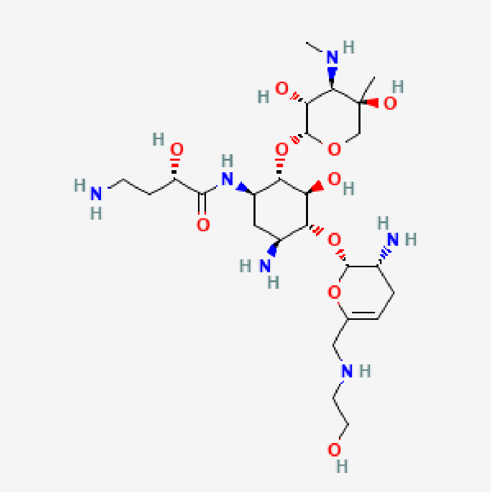

https://pubchem.ncbi.nlm.nih.gov/compound/42613186 *(accessed 24.03.23)*

###

S-7. Aztreonam - Avibactam

Aztreonam

Chemical name: aztreonam
PubChem CID: 5742832
Molecular Formula: $C_{13}H_{17}N_5O_8S_2$
Molecular Weight: 435.4g/mol
IUPAC Name: 2-[(Z)-[1-(2-amino-1,3-thiazol-4-yl)-2-[[(2S,3S)-2-methyl-4-oxo-1-sulfoazetidin-3-yl]amino]-2-oxoethylidene]amino]oxy-2-methylpropanoic acid
2D structure:

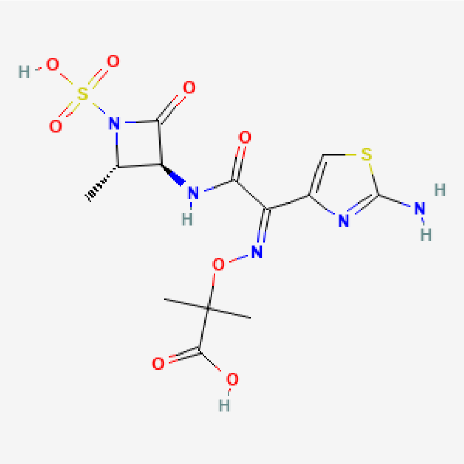

https://pubchem.ncbi.nlm.nih.gov/compound/5742832 *(accessed 24.03.23)*

Avibactam

Chemical name: avibactam
PubChem CID: 9835049
Molecular Formula: $C_7H_{11}N_3O_6S$
Molecular Weight: 265.25g/mol
IUPAC Name: [(2S,5R)-2-carbamoyl-7-oxo-1,6-diazabicyclo[3.2.1]octan-6-yl] hydrogen sulfate
2D structure:

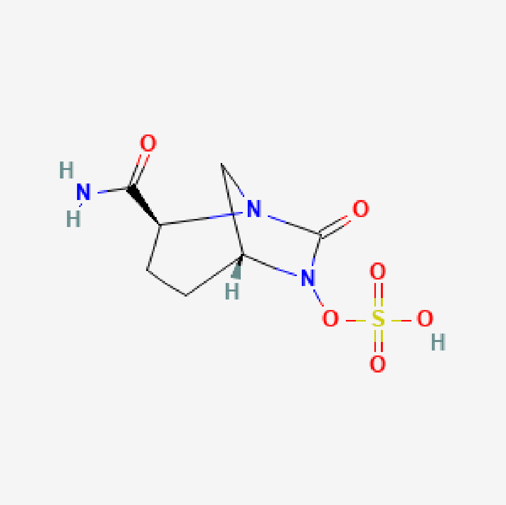

https://pubchem.ncbi.nlm.nih.gov/compound/9835049 *(accessed 24.03.23)*

###

S-8. Cefepime - Enmetazobactam
S-9. Cefepime - Zidebactam
S.10. Cefepime - Taniborbactam

Cefepime

Chemical name: cefepime
PubChem CID: 5479537
Molecular Formula: $C_{19}H_{24}N_6O_5S_2$
Molecular Weight: 480.6g/mol
IUPAC Name: (6R,7R)-7-[[(2Z)-2-(2-amino-1,3-thiazol-4-yl)-2-methoxyiminoacetyl]amino]-3-[(1-methylpyrrolidin-1-ium-1-yl)methyl]-8-oxo-5-thia-1-azabicyclo[4.2.0]oct-2-ene-2-carboxylate
2D structure:

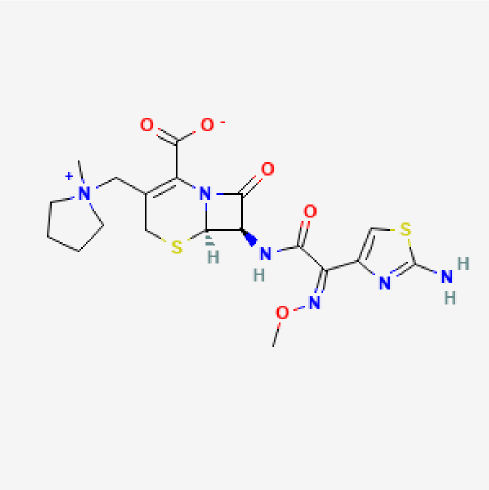

https://pubchem.ncbi.nlm.nih.gov/compound/5479537 *(accessed 24.03.23)*

Enmetazobactam

Chemical name: enmetazobactam
PubChem CID: 23653540
Molecular Formula: $C_{11}H_{14}N_4O_5S$
Molecular Weight: 314.32g/mol
IUPAC Name: (2S,3S,5R)-3-methyl-3-[(3-methyltriazol-3-ium-1-yl)methyl]-4,4,7-trioxo-4lambda6-thia-1-azabicyclo[3.2.0]heptane-2-carboxylate
2D structure:

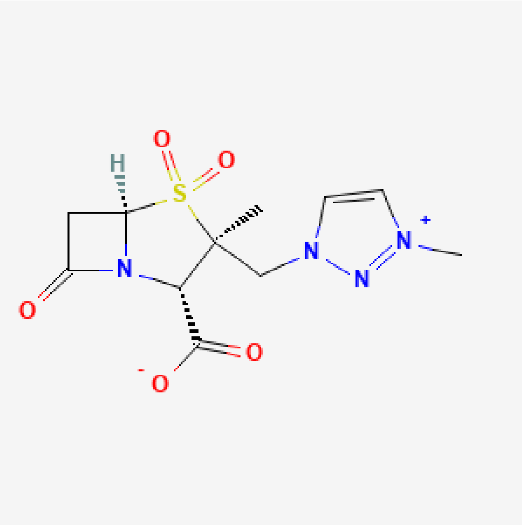

https://pubchem.ncbi.nlm.nih.gov/compound/23653540 *(accessed 24.03.23)*

Zidebactam

Chemical name: zidebactam
PubChem CID: 77846445
Molecular Formula: $C_{13}H_{21}N_5O_7S$
Molecular Weight: 391.4g/mol

IUPAC Name: [(2S,5R)-7-oxo-2-[[[(3R)-piperidine-3-carbonyl]amino]carbamoyl]-1,6-diazabicyclo[3.2.1]octan-6-yl] hydrogen sulfate
2D structure:

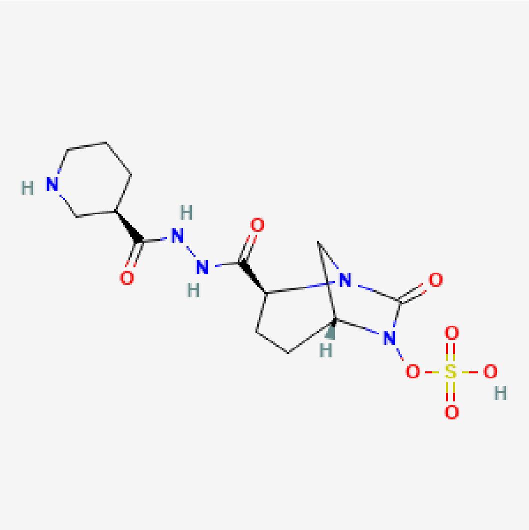

https://pubchem.ncbi.nlm.nih.gov/compound/77846445 *(accessed 24.03.23)*

Taniborbactam

Chemical name: taniborbactam
PubChem CID: 76902493
Molecular Formula: $C_{19}H_{28}BN_3O_5$
Molecular Weight: 389.3g/mol
IUPAC Name: (3R)-3-[[2-[4-(2-aminoethylamino)cyclohexyl]acetyl]amino]-2-hydroxy-3,4-dihydro-1,2-benzoxaborinine-8-carboxylic acid
2D structure:

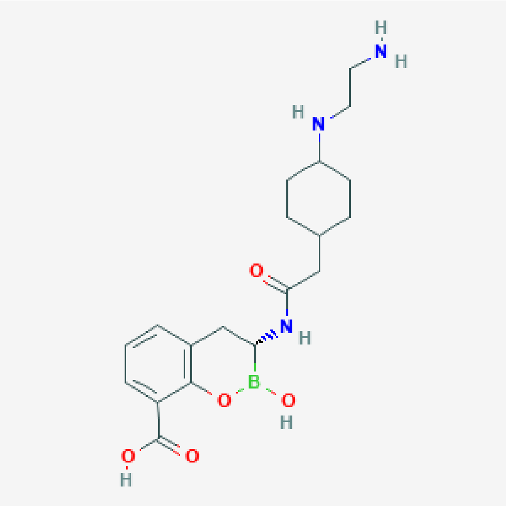

https://pubchem.ncbi.nlm.nih.gov/compound/76902493 *(accessed 24.03.23)*

###

S-11. Sulbactam - Durlobactam

Sulbactam

Chemical name: sulbactam
PubChem CID: 130313
Molecular Formula: $C_8H_{11}NO_5S$
Molecular Weight: 233.24g/mol
IUPAC Name: (2S,5R)-3,3-dimethyl-4,4,7-trioxo-4lambda6-thia-1-azabicyclo[3.2.0]heptane-2-carboxylic acid
2D structure:

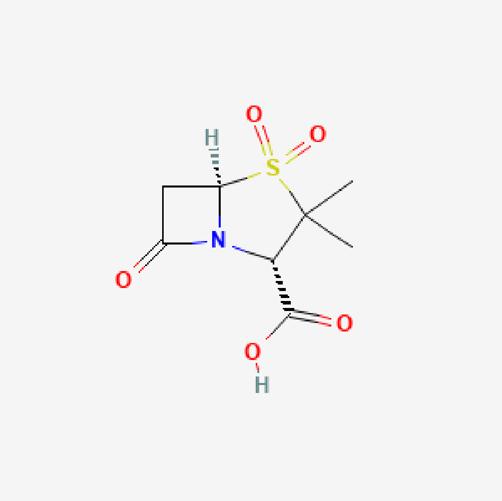

https://pubchem.ncbi.nlm.nih.gov/compound/130313 *(accessed 24.03.23)*

Durlobactam

Chemical name: durlobactam
PubChem CID: 89851852
Molecular Formula: $C_8H_{11}N_3O_6S$
Molecular Weight: 277.26g/mol
IUPAC Name: [(2S,5R)-2-carbamoyl-3-methyl-7-oxo-1,6-diazabicyclo[3.2.1]oct-3-en-6-yl] hydrogen sulfate
2D structure:

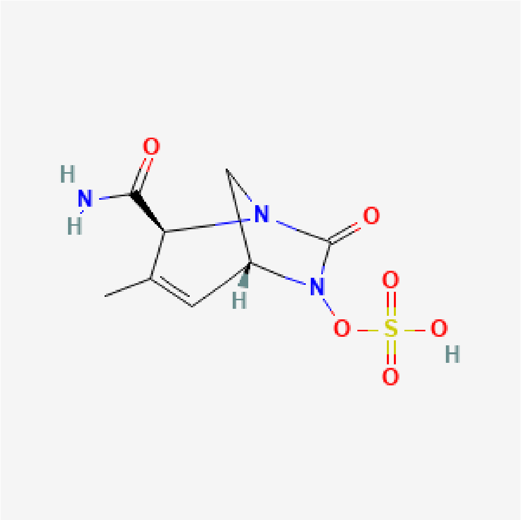

https://pubchem.ncbi.nlm.nih.gov/compound/89851852 *(accessed 24.03.23)*

###

S-12. Sulopenem

Chemical name: sulopenem
PubChem CID: 9950244
Molecular Formula: $C_{12}H_{15}NO_5S_3$
Molecular Weight: 349.5g/mol
IUPAC Name: (5R,6S)-6-[(1R)-1-hydroxyethyl]-7-oxo-3-[(1R,3S)-1-oxothiolan-3-yl]sulfanyl-4-thia-1-azabicyclo[3.2.0]hept-2-ene-2-carboxylic acid
2D structure:

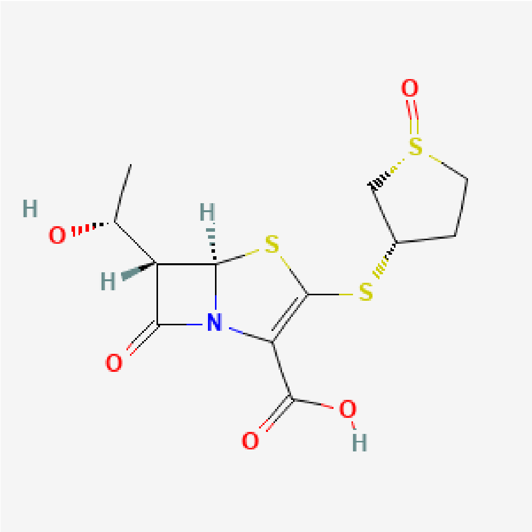

https://pubchem.ncbi.nlm.nih.gov/compound/9950244 *(accessed 24.03.23)*

###

S-13. Tebipenem

Chemical name: tebipenem
PubChem CID: 9800194
Molecular Formula: $C_{16}H_{21}N_3O_4S_2$
Molecular Weight: 383.5g/mol
IUPAC Name: (4R,5S,6S)-3-[1-(4,5-dihydro-1,3-thiazol-2-yl)azetidin-3-yl]sulfanyl-6-[(1R)-1-hydroxyethyl]-4-methyl-7-oxo-1-azabicyclo[3.2.0]hept-2-ene-2-carboxylic acid
2D structure:

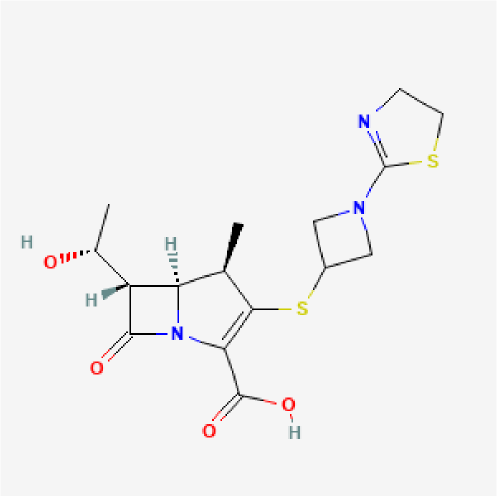

https://pubchem.ncbi.nlm.nih.gov/compound/9800194 *(accessed 24.03.23)*

###

S-14. Benapenem

Chemical name: benapenem
PubChem CID: 58326208
Molecular Formula: $C_{22}H_{28}N_4O_7S_2$
Molecular Weight: 524.6g/mol
IUPAC Name: (4R,5S,6S)-6-[(1R)-1-hydroxyethyl]-4-methyl-7-oxo-3-[(3S,5S)-5-[(4-sulfamoylphenyl)methylcarbamoyl]pyrrolidin-3-yl]sulfanyl-1-azabicyclo[3.2.0]hept-2-ene-2-carboxylic acid
2D structure:

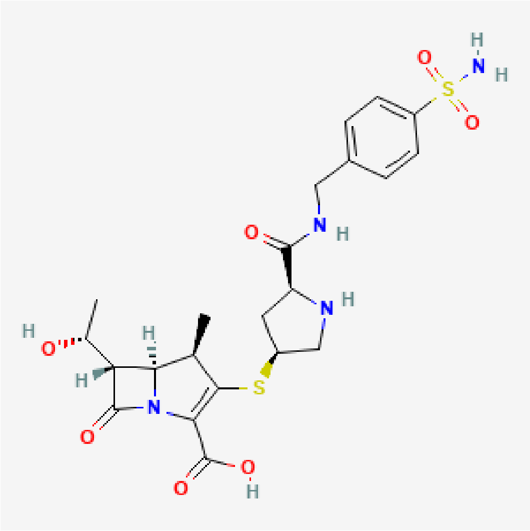

https://pubchem.ncbi.nlm.nih.gov/compound/58326208 *(accessed 24.03.23)*
